# Liquid Chromatography‐Electrospray Ionization‐Tandem Mass Spectrometry Profiling, Antioxidant, Antibacterial, and Antidiabetic Properties of *Fagonia cretica* L.: Insights From In Vitro and In Silico Approaches

**DOI:** 10.1002/open.202500592

**Published:** 2026-05-21

**Authors:** Neghmouche Nacer Salah, Elhafnaoui Lanez, Mohammed Larbi Benamor, Ouafa Zouari Ahmed, Yahia Bekkar, Moussa Senigra, Lazhar Bechki, Stefania Garzoli, Touhami Lanez

**Affiliations:** ^1^ Department of Chemistry Faculty of Exact Sciences VTRS Laboratory University of El Oued El Oued Algeria; ^2^ Laboratory of Biology, Environment, and Health Faculty of Natural and Life Sciences University of El‐Oued El‐Oued Algeria; ^3^ Laboratory of Applied Chemistry and Environment (LCAE) Chemistry Department University of Hamma Lakhdar El Oued El Oued Algeria; ^4^ Department of Biology Faculty of Natural Sciences and Life University of El Oued El Oued Algeria; ^5^ Department of Chemistry VPRS Laboratory Faculty of Mathematics and Material Sciences Kasdi Merbah University Ouargla Algeria; ^6^ Department of Chemistry and Technologies of Drug Sapienza University Rome Italy

**Keywords:** bioactivities, LC–ESI–MS/MS, molecular docking, molecular dynamics simulation, phenolic compounds

## Abstract

This study provides an integrative evaluation of the aqueous extract of *Fagonia cretica* L., a species long employed in traditional medicine but insufficiently characterized through systematic experimental and computational approaches. The phytochemical profile, established by LC–ESI–MS/MS, revealed a predominance of phenolic acids (salicylic, ferulic, sinapic, gallic), flavonoids (quercetin, luteolin, apigenin, catechin, epicatechin), and the stilbene resveratrol. Quantitative assays confirmed a substantial phenolic load and flavonoid content. Biological investigations demonstrated that the extract exerted moderate yet consistent antioxidant capacity in DPPH, ABTS, CUPRAC, and ferric assays. Antibacterial activity was more selective, with pronounced inhibition against *S. aureus* and *Micrococcus luteus*, but negligible effect on *E. coli*. Inhibition of α‐amylase was observed, indicating weak to modest metabolic benefits, though less potent than acarbose. In silico analyses reinforced these findings. ADMET predictions suggested favorable absorption and safety profiles for most constituents, with minimal risks of mutagenicity or carcinogenicity. Molecular docking identified quercetin, luteolin, and catechin as leading compounds, displaying strong binding affinities across antioxidant, antibacterial, and antidiabetic protein targets—at times surpassing reference ligands. Molecular dynamics simulations of representative complexes confirmed the stability of these interactions under physiological conditions.

## Graphical abstract

*Fagonia cretica* L.: Phytochemical investigation and exploration of bioactivity through in vitro and in silico approaches.

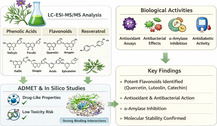

## 1 Introduction

Medicinal plants remain a cornerstone in the search for new therapeutic agents, owing to their structural diversity, bioactivity, and long‐standing ethnopharmacological relevance [1]. Among them, species of the genus *Fagonia* have drawn increasing attention, particularly in North Africa and South Asia, where they are widely used in traditional medicine for treating inflammatory disorders, infections, and metabolic diseases [2].

*Fagonia cretica* L. is one of the most studied members of this genus and is valued for its broad spectrum of biological properties. Extracts of this plant have been reported to exhibit antioxidant, antimicrobial, anti‐inflammatory, and antidiabetic activities, effects largely attributed to its phenolic acids, flavonoids, and other secondary metabolites [3]. Despite its widespread traditional use, systematic studies that integrate phytochemical profiling with both experimental assays and computational analyses remain limited, and the pharmacological potential of aqueous extracts in particular has not been fully clarified [4].

Phenolic acids and flavonoids represent the principal classes of bioactive compounds in *Fagonia* species. These metabolites are known to modulate oxidative stress, regulate enzymatic activities, and interfere with microbial growth through multiple mechanisms, including free radical scavenging, enzyme inhibition, and disruption of microbial membranes [5]. Their pharmacokinetic behavior and safety profiles further increase their relevance as potential therapeutic leads [6].

Advances in computational biology have provided powerful tools to complement traditional pharmacological assays. In silico methods, including ADMET prediction, molecular docking, and molecular dynamics simulations, allow the rapid assessment of pharmacokinetic suitability, interaction potentials, and structural stability of plant‐derived metabolites within biological targets [7]. When integrated with experimental assays, these approaches provide a more complete understanding of plant extracts and their therapeutic promise.

The present study aimed to investigate the phytochemical composition, biological activities, and in silico interactions of the aqueous extract of *F. cretica*. Specifically, the work focused on (i) profiling phenolic constituents using LC–ESI–MS/MS; (ii) evaluating antioxidant, antibacterial, and α‐amylase inhibitory activities through in vitro assays; and (iii) assessing pharmacokinetics, docking interactions, and dynamic stability of major metabolites against relevant protein targets. This integrative strategy was designed to bridge traditional use with mechanistic insights, thereby contributing to the rational development of *F. cretica* as a source of bioactive agents. Although several studies have investigated the biological activities of *Fagonia cretica*, no data are currently available on the phytochemical composition of *F. cretica* collected from the Touggourt region (Algerian Sahara), particularly using LC–ESI–MS/MS. Moreover, aqueous extracts from this population have not been previously characterized or mechanistically explored through integrated in vitro and in silico approaches.

## 2 Material and Methods

### 2.1 Chemicals

All chemicals used in this study were of analytical grade and purchased primarily from Sigma–Aldrich (Taufkirchen, Germany) and Honeywell (USA). Antioxidant assay reagents included DPPH (2,2‐diphenyl‐1‐picrylhydrazyl), ABTS (2,2′‐azinobis [3‐ethylbenzothiazoline‐6‐sulfonic acid] diammonium salt), quercetin, butylated hydroxyanisole (BHA), α‐tocopherol (TCP), and Trolox (TX). Acarbose was used as a standard inhibitor in enzymatic assays. Dimethyl sulfoxide (DMSO) was employed for dissolving specific reagents when required. For LC–ESI–MS/MS analysis, seventeen phenolic reference standards were obtained from Sigma–Aldrich (Merck, Germany) with purities ≥95%, including shikimic acid, gallic acid, chlorogenic acid, hydroxybenzaldehyde, caffeic acid, vanillin, p‐coumaric acid, salicylic acid, polydatin, trans‐ferulic acid, scutellarin, isoquercitrin, coumarin, rutin, fisetin, quercetin, and kaempferol.

### 2.2 Plant Material

The aerial parts of *Fagonia cretica* L. were collected during the flowering stage in June 2023 from Touggourt Province, Algeria (33°22′ N, 6°51′ E). The collection complied with institutional and national guidelines for plant use. Botanical authentication was performed by Professor Chouikh A., Department of Life and Nature Sciences, Faculty of Sciences, University of Hama Lakhder El‐Oued. Taxonomic identification was confirmed using the Flora of Algeria [8] and by comparison with previously published descriptions [9]. A voucher specimen (FC‐85) was prepared and deposited in the departmental herbarium for future reference. This work represents the first phytochemical and biological investigation of *Fagonia cretica* collected from the Touggourt region.

### 2.3 Extracts Preparation

The aerial parts of *Fagonia cretica* L. were washed, shade‐dried at room temperature, and powdered using a mechanical grinder. The aqueous extract was prepared following a modified protocol of Zahnit et al. [10]. Briefly, 50 g of powdered material was macerated in 500 mL of deionized water, heated at 70°C for 60 min under constant stirring, and subsequently incubated at room temperature for 48 h in the dark with intermittent agitation. The mixture was filtered, concentrated under reduced pressure at 50°C (40 rpm) using a rotary evaporator, and stored in amber vials at 4°C until further analysis.

### 2.4 Quantification of Phenolic Compounds

#### 2.4.1 Total Polyphenol Content

The total polyphenol content was determined using the Folin–Ciocalteu colorimetric assay as described by Tlili et al. [11], with minor modifications. Gallic acid was used to construct the calibration curve, and results were expressed as micrograms of gallic acid equivalents per milligram of extract (µg GAE/mg E). Absorbance was recorded with a Multiskan SkyHigh Microplate Spectrophotometer (Thermo Fisher Scientific, USA).

#### 2.4.2 Total Flavonoid Content

The total flavonoid content was assessed using the aluminum chloride colorimetric method originally reported by Segueni et al. [12], with modifications. Quercetin was used to prepare the calibration curve, and results were expressed as micrograms of quercetin equivalents per milligram of extract (µg QE/mg E). All measurements were carried out using a Multiskan SkyHigh Microplate Spectrophotometer (Thermo Fisher Scientific, USA).

### 2.5 Profiling of Extract Using LC–ESI–MS/MS

The phytochemical profile of the aqueous extract of *Fagonia cretica* L. was determined using LC–ESI–MS/MS analysis, following the protocol of Boudebia et al. [13] with modifications. Chromatographic separation was performed on an Agilent Poroshell 120 EC‐C18 column (100 mm × 3.0 mm, 2.7 µm) under reversed‐phase conditions. An Agilent 1260 Infinity II liquid chromatography system coupled to a tandem mass spectrometer (LC–MS/MS) was used for compound identification and quantification.

Sample preparation involved dissolving the dried aqueous extract in methanol (50 mg/mL), followed by filtration through a 0.22 µm membrane filter. A 5 µL aliquot was injected for analysis. The mobile phase consisted of water (A) and methanol (B), both containing 0.1% formic acid and 5 mM ammonium formate. The gradient program was: 0–1 min, 3% B; 1–3 min, 25% B; 4–12 min, 50% B; 13–21 min, 90% B; and 22–25 min, re‐equilibration at 3% B. The column temperature was maintained at 40°C.

Mass spectrometry parameters were: capillary voltage 4000 V, nitrogen flow 11 L/min, nebulizer pressure 15 psi, and source temperature 300°C. Quantification was based on calibration curves from authentic phenolic standards. Method validation, including linearity (LR), limit of detection (LOD), and limit of quantification (LOQ), was performed according to Yilmaz (2020) [14].

### 2.6 LC–ESI–MS/MS Method Validation and Quantitative Analysis

The LC–ESI–MS/MS method was validated for quantitative analysis following commonly accepted analytical guidelines for targeted LC–MS/MS assays. Calibration curves were constructed using authentic phenolic standards at six concentration levels and showed good linearity within the tested ranges, with coefficients of determination (*R* ^2^) ≥ 0.995.

Limits of detection (LOD) and quantification (LOQ) were determined based on signal‐to‐noise ratios of approximately 3 and 10, respectively. Method accuracy was evaluated by recovery experiments at three concentration levels (low, medium, high) by spiking known amounts of standards into the extract matrix. Precision was assessed as intra‐day and inter‐day repeatability and expressed as relative standard deviation (RSD%).

Matrix effects were evaluated by comparing the slopes of calibration curves prepared in solvent with those obtained in post‐extraction spiked samples and expressed as percentage matrix effect.

Quantification of metabolites in the aqueous extract was performed using external calibration curves, and results are expressed as mg per g of dry extract (mg/g) (mean ± SD, *n* = 3).

### 2.7 Evaluation of In Vitro Antioxidant Activities

The antioxidant activity of the aqueous extract of *Fagonia cretica* L. was assessed through four complementary assays: DPPH, ABTS, cupric reducing antioxidant capacity (CUPRAC), and ferric reducing power. Standard antioxidants included quercetin (QCT), ascorbic acid (AsA), α‐tocopherol (TCP), Trolox (TX), and butylated hydroxyanisole (BHA).

The percentage inhibition was calculated using the following Equation (1):

(1) $$\text{Inhibition}\left(\%\right)=\frac{A_{\text{control}}-A_{\text{sample}}}{A_{\text{control}}}\times 100$$

where *A*_control_ is the absorbance of the control (without extract) and *A*_sample_ is the absorbance in the presence of extract. IC_50_ values (µg/mL), representing the concentration required to inhibit 50% of the radical or reduce 50% of the oxidant, were determined from dose–response curves.

#### 2.7.1 DPPH Radical Scavenging Assay

The ability of the extract to scavenge DPPH radicals was determined according to Lanez et al. [15]. Briefly, 1 mL of various concentrations of the extract was mixed with 1 mL of 0.1 mM DPPH solution in methanol. After incubation in the dark at room temperature for 30 min, the absorbance was recorded at 517 nm. The IC_50_ value was calculated from the inhibition curve.

#### 2.7.2 ABTS Radical Cation Scavenging Assay

ABTS radical scavenging activity was assessed following the procedure of Adaika et al. [16]. ABTS•^+^ solution was prepared by mixing 7 mM ABTS with 2.45 mM potassium persulfate and incubating for 16 h in the dark. The solution was diluted to an absorbance of 0.70 ± 0.02 at 734 nm. Then, 1 mL of extract at different concentrations was mixed with 1 mL of ABTS•^+^ solution, and absorbance was measured after 6 min at 734 nm. IC_50_ values were determined.

#### 2.7.3 Cupric Reducing Antioxidant Capacity (CUPRAC)

CUPRAC activity was evaluated according to Loucif et al. [17]. The reaction mixture consisted of 1 mL of 10 mM CuCl_2_, 1 mL of 7.5 mM neocuproine, 1 mL of 1 M ammonium acetate buffer (pH 7.0), and 0.5 mL of extract at various concentrations. The total volume was adjusted to 4.1 mL with distilled water. After incubation at room temperature for 30 min, absorbance was measured at 450 nm. Results were expressed as A_0_._5_ (µg/mL), defined as the concentration required to achieve 50% of maximal absorbance.

#### 2.7.4 Ferric Reducing Power Assay

The reducing power was determined according to Chouikh et al. [18]. Extract solutions (1 mL) were mixed with 2.5 mL of 200 mM phosphate buffer (pH 6.6) and 2.5 mL of 1% potassium ferric. The mixture was incubated at 50°C for 20 min, and then 2.5 mL of 10% trichloroacetic acid was added, followed by centrifugation (3000 rpm, 10 min). The supernatant (2.5 mL) was mixed with 2.5 mL distilled water and 0.5 mL of 0.1% FeCl_3_, and absorbance was measured at 700 nm. Results were expressed as *A*_0_._5_ values.

### 2.8 Antibacterial Activity

#### 2.8.1 Bacterial Strains

The antibacterial activity of the aqueous extract of *Fagonia cretica* L. was evaluated against a panel of clinically relevant reference strains obtained from the Microbiology Laboratory, University of El Oued, Algeria. The tested microorganisms included Gram‐positive bacteria (*Bacillus subtilis* ATCC 9372, *Micrococcus luteus* ATCC 4698, *Staphylococcus aureus* ATCC 223) and Gram‐negative bacteria (*Escherichia coli* ATCC 25922, *Pseudomonas aeruginosa* ATCC 27853, *Enterobacter cloacae* ATCC 13047).

#### 2.8.2 Agar Well Diffusion Assay

Preliminary antibacterial screening was performed using the agar well diffusion method. Mueller–Hinton agar plates were inoculated with standardized bacterial suspensions (≈10^8^ CFU/mL). Wells (8 mm diameter) were aseptically created and filled with 25 µL of the extract (200 mg/mL in 5% DMSO). Plates were pre‐incubated at 4°C for 1 h to promote diffusion, followed by incubation at 37°C for 24 h. Antibacterial activity was determined by measuring the inhibition zone diameters. Ciprofloxacin (CIP), vancomycin (VN), cefalexin (CN), and imipenem (IMP) served as positive controls. A 5% (v/v) DMSO solution was used as the negative control and produced no detectable inhibition zones (0 mm) against any of the tested bacterial strains.

#### 2.8.3 Minimum Inhibitory Concentration (MIC) and Minimum Bactericidal Concentration (MBC)

MIC and MBC values were determined by the broth microdilution method in 96‐well plates, adapted from Damtie and Mekonnen [19]. Serial twofold dilutions of the extract (200–0.78 mg/mL in 5% DMSO) were prepared. Gentamicin was included as a positive control (1.25–0.019 mg/mL). After incubation at 37°C for 24 h, MIC was defined as the lowest concentration preventing visible growth. To determine MBC, aliquots from non‐turbid wells were streaked onto fresh Mueller–Hinton agar plates and incubated under identical conditions. The lowest concentration producing no bacterial colony growth was recorded as the MBC. The negative control (5% DMSO) showed no inhibitory or bactericidal effect at any tested concentration, confirming that the observed antibacterial activity was attributable solely to the plant extract.

### 2.9 In Vitro α‐Amylase Inhibition Assay

The inhibitory effect of the aqueous extract of *Fagonia cretica* L. on α‐amylase activity was evaluated using the Lugol's iodine method described by Cherrada et al. [20], with slight modifications. The reaction mixture contained varying concentrations of the extract and soluble starch substrate. Following incubation, iodine solution was added, and residual starch was quantified spectrophotometrically at 620 nm. Acarbose, a clinically approved α‐amylase inhibitor, was used as the positive control. The percentage inhibition was calculated relative to the uninhibited control, and IC_50_ values were derived from dose–response curves.

### 2.10 In Silico Studies

#### 2.10.1 Ligand Preparation

Secondary metabolites identified by LC–ESI–MS/MS analysis of *Fagonia cretica* L. were retrieved from the PubChem database [21] in SDF format. Structures were converted to PDB format using Open Babel [22] and optimized by geometry minimization with the Merck Molecular Force Field (MMFF94) in Avogadro v1.2.0 [23, 24]. Optimized ligands were subsequently used for ADMET screening and docking.

#### 2.10.2 ADMET and Drug‐Likeness Prediction

Twenty top metabolites were screened for pharmacokinetic and toxicity properties using SwissADME [25], pkCSM [26], and admetSAR [27]. Parameters included absorption (intestinal permeability, P‐glycoprotein interactions), distribution (blood–brain barrier penetration, plasma protein binding), metabolism (CYP450 inhibition), excretion, and toxicity endpoints (mutagenicity, carcinogenicity, hepatotoxicity). Based on drug‐likeness and ADMET scores, the ten best‐performing compounds were selected for molecular docking.

#### 2.10.3 Target Protein Preparation

Protein targets were selected to complement the in vitro assays: antioxidant enzymes (catalase, PDB ID: 1DGF; superoxide dismutase, PDB ID: 1CBJ; glutathione reductase, PDB ID: 1XAN), antibacterial enzymes (DNA gyrase B, PDB ID: 6RKS; dihydrofolate reductase, PDB ID: 1RX2; TEM‐1 β‐lactamase, PDB ID: 1ZG4), and the antidiabetic enzyme human pancreatic α‐amylase (PDB ID: 1HNY). Crystal structures were obtained from the RCSB Protein Data Bank [28]. Proteins were pre‐processed in AutoDockTools v1.5.7 [29] by removing water molecules and co‐crystallized ligands, adding polar hydrogens, and assigning Kollman charges. Receptors were kept rigid, while ligands remained flexible.

#### 2.10.4 Molecular Docking Protocol

Docking simulations were performed using AutoDock Vina v1.2.7 [30]. The docking grid was centered on the co‐crystallized ligand or active site, with dimensions sufficient to cover the binding pocket plus a 5–6 Å margin. Parameters included exhaustiveness = 16, num_modes = 20, and energy range = 3 kcal·mol^−1^. The top 20 poses per ligand were retained. Protein–ligand interactions were analyzed using Protein–Ligand Interaction Profiler (PLIP) [31] and visualized in PyMOL v3.1 [32] and Discovery Studio Visualizer (DSV) [33].

#### 2.10.5 Docking Validation

The docking protocol was validated by re‐docking the native co‐crystallized ligands into their binding sites. Root‐mean‐square deviation (RMSD) values between docked and experimental poses were calculated in PyMOL, with ≤2.0 Å accepted as reliable [34]. Only validated parameters were used for the docking of ADMET‐filtered compounds.

#### 2.10.6 Molecular Dynamics Simulations

To assess stability and conformational behavior of the most promising docked complexes, molecular dynamics (MD) simulations were carried out in GROMACS v2023 [35]. The CHARMM36 force field [36] was applied for proteins, while ligand parameters were generated using CGenFF [37]. Each protein–ligand complex was solvated in a triclinic box with TIP3P water molecules, maintaining at least 1.0 nm from the solute to the box edge. Counterions (Na^+^/Cl^−^) were added for neutralization. Energy minimization was performed with the steepest descent algorithm until reaching a maximum force < 1000 kJ·mol^−1^·nm^−1^.

Equilibration was performed in two steps: (i) NVT ensemble for 100 ps at 300 K using the V‐rescale thermostat, followed by (ii) NPT ensemble for 100 ps at 1 bar using the Parrinello–Rahman barostat. Production runs of 300 ns were performed with a 2 fs integration step, applying periodic boundary conditions and the Particle Mesh Ewald (PME) method for long‐range electrostatics. Trajectory analyses included root–mean‐square deviation (RMSD), root–mean‐square fluctuation (RMSF), radius of gyration (Rg), and solvent‐accessible surface area (SASA), providing insights into structural stability and compactness.

#### 2.10.7 MM‐PBSA Binding Free Energy Calculations

Binding free energy calculations were performed using the Molecular Mechanics–Poisson–Boltzmann Surface Area (MM‐PBSA) approach to quantitatively evaluate protein–ligand interactions following molecular dynamics simulations. MM‐PBSA analysis was conducted using the gmx_MMPBSA tool on equilibrated MD trajectories [38].

Trajectories were extracted from the last 100 ns of each 300 ns production run to ensure structural stability. Snapshots were collected every 100 ps, yielding 1000 frames per system. The binding free energy (ΔG_bind) was calculated according to the equation [39]:

$$\Delta G_{\mathrm{bind}}=\Delta E_{\mathrm{MM}}+\Delta G_{\mathrm{solv}}-T\Delta S$$

where Δ*E*_MM_ includes van der Waals (Δ*E*_vdW_) and electrostatic (Δ*E*_elec_) contributions and Δ*G*_solv_ comprises polar (Δ*G*_polar_) and non‐polar (Δ*G*_nonpolar_) solvation energies. Entropic contributions (−*T*Δ*S*) were not explicitly calculated, as the analysis aimed at comparative binding trends rather than absolute free energies.

MM‐PBSA calculations were applied to selected representative complexes, including quercetin–catalase, catechin–DNA gyrase B, and quercetin–α‐amylase.

### 2.11 Statistical Analysis

All experimental assays were performed in triplicate (*n* = 3 independent measurements), and results are expressed as mean ± standard deviation (SD). Prior to applying one‐way analysis of variance (ANOVA), the assumptions of normality and homogeneity of variances were evaluated. Normality of data distribution was assessed using the Shapiro–Wilk test**,** while homogeneity of variances was examined using Levene's test.

When the assumptions were met, one‐way ANOVA followed by Tukey's honestly significant difference (HSD) post hoc test was applied to compare group means. Statistical significance was set at *p* < 0.05.

In addition, 95% confidence intervals (CI) were calculated for IC_50_ and A_0_._5_ values derived from dose–response curves to provide an estimate of parameter uncertainty. All statistical analyses were performed using XLSTAT 2016 (Addinsoft, USA).

## 3 Results and Discussion

### 3.1 Extraction Yield and Phytochemical Content

#### 3.1.1 Extraction Yield

In the present study, the bioactive constituents of *Fagonia cretica* L. aerial parts were extracted exclusively using deionized water to obtain the aqueous extract. The extraction yield, expressed as a percentage of the initial dry plant material, reached 12.45 ± 0.28%, reflecting the high solubility of polar compounds in water (Table 1).

**Table 1:** Extraction yield of *Fagonia cretica* L. aerial parts.

Sample	Solvent	Yield (% w/w)
*F. cretica* L	Water	12.45 0.28

Extraction represents a critical step in phytochemical investigations, as it selectively isolates biologically active components while removing inert or non‐essential materials [40]. The efficiency of an extraction is directly influenced by the solvent's polarity, which governs the solubility of different classes of compounds [41]. Polar solvents, such as water, are particularly effective in solubilizing phenolics, flavonoids, glycosides, and other hydrophilic metabolites, which are typically responsible for antioxidant, antibacterial, and enzyme inhibitory activities [42].

The relatively high yield observed for the aqueous extract of *F. cretica* L. is consistent with the solvent's ability to dissolve a wide spectrum of polar metabolites, as previously reported in related studies on *Fagonia* species [43, 44]. This efficiency underlines the rationale for selecting water as the extraction medium, particularly when targeting bioactive compounds relevant for antioxidant, antibacterial, and antidiabetic applications.

#### 3.1.2 Total Phenolic Content (TPC) and Total Flavonoid Content (TFC)

Polyphenols and flavonoids are major contributors to the biological activities of plant extracts, including antioxidant, antibacterial, and enzyme inhibitory effects. In the present study, the aqueous extract of *Fagonia cretica* L. was analyzed for its total polyphenol content (TPC) and total flavonoid content (TFC) (Table 2).

**Table 2:** Total polyphenols and flavonoids in the aqueous extract of *Fagonia cretica* L.

Extract	TPC (µg GAE/mg extract)	TFC (µg QE/mg extract)
*F. cretica* L	118.72 ± 0.41	46.85 ± 0.53

The aqueous extract exhibited a TPC of 118.72 ± 0.41 µg GAE/mg extract and a TFC of 46.85 ± 0.53 µg QE/mg extract, reflecting the efficient extraction of polar phenolic and flavonoid compounds. These values are consistent with the known tendency of polar solvents, such as water, to solubilize hydrophilic secondary metabolites. In contrast, nonpolar solvents typically yield lower phenolic and flavonoid contents due to their poor solubilizing capacity [45, 46].

The aqueous extract exhibited a TPC of 118.72 ± 0.41 µg GAE/mg extract and a TFC of 46.85 ± 0.53 µg QE/mg extract, reflecting the effective extraction of polar phenolic and flavonoid compounds. These values are consistent with the known tendency of polar solvents, such as water, to solubilize hydrophilic secondary metabolites, whereas nonpolar solvents generally yield lower phenolic and flavonoid contents due to poor solubility [45, 46].

### 3.2 Phenolic Profile Characterization by LC–ESI–MS/MS

Plant phenolics and flavonoids constitute one of the most important classes of secondary metabolites in medicinal plants and are widely recognized as key contributors to antioxidant, antimicrobial, and enzyme inhibitory activities [38]. In the present study, the phenolic composition of the aqueous extract of *Fagonia cretica* L. collected from the Touggourt region (Algerian Sahara) was investigated using a validated LC–ESI–MS/MS method, allowing both qualitative identification and quantitative determination of major constituents.

LC–ESI–MS/MS analysis enabled the identification of 20 phenolic and related compounds, belonging mainly to phenolic acids, flavones, flavonols, flavan‐3‐ols, flavanones, stilbenes, and triterpenoids. The chromatographic retention times, mass transitions, and relative signal intensities are summarized in Table 3, while the full chromatogram and extended metabolite list are provided in Figure S1 and Table S1.

**Table 3:** Major phytochemicals identified in the aqueous extract of *Fagonia cretica* L. by LC–ESI–MS/MS.

ID	Compound	Formula	m/z (transition)	Ret. time, min	Peak area	Class
34	Salicylic acid	C_7_H_6_O_3_	137.2 → 92.9	7.11	4,015,746	Phenolic acid
16	Ferulic acid	C_10_H_10_O_4_	195.0 → 44.7	5.82	1,620,532	Phenolic acid
10	Apigenin	C_15_H_10_O_5_	271.1 → 152.9	5.96	908,707	Flavone
24	Sinapic acid	C_11_H_12_O_5_	225.0 → 207.1	6.89	794,651	Phenolic acid
1	Catechin	C_15_H_14_O_6_	291.1 → 161.1	8.04	161,695	Flavan‐3‐ol
4	Myricetin	C_15_H_10_O_8_	318.5 → 256.0	7.03	226,714	Flavonol
20	Quercetin	C_15_H_10_O_7_	303.0 → 153.1	6.54	19,212	Flavonol
21	Resveratrol	C_14_H_12_O_3_	229.0 → 107.1	6.88	299,001	Stilbene
15	Epicatechin	C_15_H_14_O_6_	290.8 → 123.1	7.04	42,897	Flavan‐3‐ol
14	Curcumin	C_21_H_20_O_6_	368.9 → 177.0	6.90	44,733	Diarylhept.
22	Rutin	C_27_H_30_O_16_	611.0 → 303.1	6.74	2,691	Flavonol gly.
18	Oleanolic acid	C_30_H_48_O_3_	457.3 → 81.0	7.90	105,926	Triterpenoid
17	Luteolin	C_15_H_10_O_6_	286.7 → 153.0	6.68	9,474	Flavone
12	Methoxybenzoic acid	C_8_H_8_O_3_	153.0 → 135.0	6.66	181,233	Phenolic acid
26	Kojic acid	C_6_H_6_O_4_	142.7 → 69.0	7.02	31,015	Hydroxyacid
27	Naringenin	C_15_H_12_O_5_	273.0 → 153.0	6.54	41,441	Flavanone
30	Caffeic acid	C_9_H_8_O_4_	179.1 → 135.0	6.75	33,268	Phenolic acid
31	Gallic acid	C_7_H_6_O_5_	169.1 → 125.0	1.27	6,128	Phenolic acid
29	Vanillin	C_8_H_8_O_3_	153.0 → 64.7	6.88	43,685	Aromatic alde.
33	p‐Coumaric acid	C_8_H_8_O_4_	163.0 → 118.9	6.47	16,658	Phenolic acid

To ensure analytical robustness, the LC–ESI–MS/MS method was fully validated, and performance parameters including linearity, limits of detection and quantification, recovery, repeatability (RSD%), and matrix effects are reported in Table S2. Quantification was performed using external calibration with authentic standards, and the results are expressed as absolute concentrations (mg/g dry extract) for the major metabolites (Table S3), rather than peak area alone.

Among the identified compounds, phenolic acids predominated quantitatively, with salicylic acid, ferulic acid, and sinapic acid representing the most abundant constituents, followed by flavonoids such as apigenin and catechin. Flavonols (quercetin, myricetin), flavan‐3‐ols (catechin, epicatechin), the stilbene resveratrol, and minor triterpenoid components (oleanolic acid) were also detected at lower concentrations. This compositional profile reflects the preferential extraction of polar phenolics using water as solvent.

To the best of our knowledge, this is the first LC–ESI–MS/MS‐based phytochemical characterization of *Fagonia cretica* from the Touggourt region. The predominance of salicylic acid, ferulic acid, apigenin, and sinapic acid highlights a distinct phenolic fingerprint that differs from previously reported profiles of Fagonia species collected from other regions. These differences may be attributed to geographical, climatic, and ecological factors, supporting the existence of a region‐specific chemotype*.*

The dominant constituents included salicylic acid (peak area 4,015,746), ferulic acid (1,620,532), apigenin (908,707), and sinapic acid (794,651), highlighting the predominance of phenolic acids and flavones in the extract. Additional compounds of interest were catechin, myricetin, quercetin, resveratrol, epicatechin, rutin, and oleanolic acid, all of which are extensively documented for their pharmacological relevance [47].

These findings are consistent with earlier phytochemical investigations of *Fagonia* species, where flavonoids such as quercetin, rutin, and catechin were reported as key metabolites [48, 49]. However, the notably high abundance of salicylic and ferulic acids in the present Touggourt population of *F. cretica* suggests a distinct chemotype, distinguishing it from other regional species. Similar variations have been attributed to differences in extraction solvent, plant organ, and ecological conditions [50].

The pharmacological relevance of the identified metabolites provides a strong rationale for the biological activities of *F. cretica*. For instance, salicylic acid is known for antifungal and antibacterial properties [51], whereas ferulic and sinapic acids act as potent antioxidants protecting against oxidative stress–mediated pathologies [52]. Flavonoids such as apigenin and quercetin exhibit strong free‐radical scavenging, antimicrobial effects, and digestive enzyme inhibition [53]. Furthermore, the presence of resveratrol, a bioactive stilbene with antioxidant and anti‐inflammatory activity, reinforces the therapeutic potential of the extract.

In recent years, there has been increasing evidence that plant extracts and their nanoformulations can exert multiple biological activities beyond those investigated here, including anticancer, antidiabetic, antioxidant, and antimicrobial effects. For example, green‐synthesized silver nanoparticles using plant extracts have been reported to exhibit anticancer efficacy via modulation of reactive oxygen species and induction of apoptotic pathways in tumor models, underscoring the broad therapeutic potential of plant‐mediated nanoparticle systems [54]. Moreover, nanoformulations of plant bioactives are increasingly studied as delivery systems to overcome limitations of crude extracts, such as poor solubility and bioavailability. Nano‐based delivery of phytochemicals has been shown to enhance antidiabetic effects in vitro and in vivo by improving pharmacokinetic profiles and target specificity relative to native plant products [55, 56].

### 3.3 In Vitro Biological Activities

#### 3.3.1 Antioxidant Activity

Table 4 summarizes the antioxidant activities of the aqueous extract of *Fagonia cretica* L. compared with standard antioxidants (quercetin, butylated hydroxyanisole, α‐tocopherol, Trolox, and ascorbic acid). Activity was expressed as IC_50_ values for radical scavenging assays (DPPH and ABTS) and as A_0_._5_ values for electron‐transfer–based assays (CUPRAC and ferric reducing power).

**Table 4:** In vitro antioxidant activity of the aqueous extract of *Fagonia cretica* L. compared with standards.

Sample	DPPH IC_50_, µg/mL	95% CI	ABTS IC_50_, µg/mL	95% CI	CUPRAC A_0.5_, µg/mL	95% CI	Ferric A_0.5_, µg/mL	95% CI
*F. cretica* (Aq)	185.6 ± 1.1	178.9–192.3	92.8 ± 0.9	87.6–98.0	86.9 ± 1.3	79.1–94.7	99.7 ± 1.2	92.7–106.7
Quercetin	3.5 ± 0.2	2.6–4.4	<1.6	—	2.1 ± 0.0	—	5.4 ± 0.2	4.5–6.3
BHA	9.5 ± 0.1	8.9–10.1	8.0 ± 0.1	7.4–8.6	—	—	1.8 ± 0.1	1.3–2.3
α‐Tocopherol	6.7 ± 0.1	6.1–7.3	—	—	17.0 ± 0.4	14.8–19.2	7.7 ± 1.3	2.3–13.1
Trolox	4.1 ± 0.2	3.2–5.0	2.8 ± 0.1	2.2–3.4	4.9 ± 0.2	4.0–5.8	3.8 ± 0.1	3.2–4.4

Values are expressed as mean ± SD (*n* = 3). Normality (Shapiro–Wilk) and homogeneity of variance (Levene's test) were verified prior to one‐way ANOVA. Confidence intervals (95% CI) were calculated for IC_50_ and A_0_._5_ values.

The aqueous extract of *F. cretica* exhibited weak antioxidant potential across all assays. In the DPPH radical scavenging assay, the extract showed an IC_50_ of 185.6 ± 1.1 µg/mL, weaker than quercetin (3.5 ± 0.2 µg/mL) and BHA (9.5 ± 0.1 µg/mL), but within the typical range for aqueous fractions of medicinal plants. A similar pattern appeared in the ABTS assay, where the extract recorded an IC_50_ of 92.8 ± 0.9 µg/mL, confirming its ability to neutralize both nitrogen‐ and oxygen‐centered radicals. For CUPRAC and ferric assays, the extract produced A_0_._5_ values of 86.9 ± 1.3 and 99.7 ± 1.2 µg/mL, respectively, supporting its modest reducing power.

These results indicate consistent, though modest, antioxidant activity relative to standards. The extract's polyphenolic content, as determined by LC–MS/MS, strongly correlates with its biological performance. Compounds such as gallic, ferulic, caffeic, and sinapic acids, along with flavonoids including quercetin, rutin, apigenin, and catechin, are well‐documented contributors to radical scavenging and redox properties [52]. For example, quercetin and rutin possess catechol groups enabling hydrogen atom transfer and transition‐metal chelation, while chlorogenic and caffeic acids exhibit strong electron‐donating capacities [57]. The detection of resveratrol further strengthens the antioxidant profile, given its dual free‐radical scavenging and anti‐inflammatory roles. Comparisons with related studies confirm that solvent polarity critically influences antioxidant recovery. Nayila et al. reported that methanolic extracts of *Fagonia indica* displayed markedly stronger DPPH activity than aqueous fractions, a trend also seen in *F. cretica* here [58]. Similarly, *F. arabica* and *F. bruguieri* extracts prepared with organic solvents yielded higher antioxidant activity than their aqueous counterparts [59]. Nonetheless, aqueous extracts remain valuable due to their safety, accessibility, and the presence of water‐soluble phenolics that may exert synergistic effects despite lower potency.

#### 3.3.2 Antibacterial Activity

The antibacterial activity of the aqueous extract of *F. cretica* L. was tested against six reference bacterial strains, including three Gram‐positive (*Bacillus subtilis*, *Staphylococcus aureus*, *Micrococcus luteus*) and three Gram‐negative (*Escherichia coli*, *Pseudomonas aeruginosa*, *Enterobacter cloacae*). Inhibition zone diameters are shown in Table 5, while MIC and MBC values are summarized in Tables 6 and 7.

**Table 5:** Antibacterial activity of the aqueous extract of *Fagonia cretica* L. (agar well diffusion).

Bacterial strains		*B. subtilis*	*S. aureus*	*E. cloacae*	*E. Coli*	*P. aeruginosa*	*M. luteus*
***Inhibition zone diameter,* *mm***							
*Fagonia cretica* L.	Aqueous extract	11 ± 0.5	13 ± 0.6	10 ± 0.5	NA	12 ± 0.7	9 ± 0.0
Positive control	VN	15 ± 0.6	NT	NT	NT	NT	16 ± 0.0
	CIP	NT	NT	NT	NT	22 ± 0.7	NT
	CN	NT	NT	18 ± 0.1	NT	NT	NT
	IMP	NT	16 ± 0.9	NT	20 ± 0.0	NT	NT
Negative control	DMSO 5%	0 ± 0.0	0 ± 0.0	0 ± 0.0	0 ± 0.0	0 ± 0.0	0 ± 0.0

Abbreviations: NA, no activity; NT, not tested.

**Table 6:** Minimum inhibitory concentrations (MIC) of *Fagonia cretica* L. aqueous extract.

Bacterial strains	*B. subtilis*	*S. aureus*	*E. cloacae*	*P. aeruginosa*	*M. luteus*
*Fagonia cretica* L.	12.5 ± 0.09	6.25 ± 0.11	25 ± 0.00	12.5 ± 0.08	6.25 ± 0.07
Gentamicin	<0.019	<0.019	<0.019	<0.019	<0.019

**Table 7:** Minimum bactericidal concentrations (MBC) of *Fagonia cretica* L. aqueous extract.

Bacterial strains	*B. subtilis*	*S. aureus*	*E. cloacae*	*P. aeruginosa*	*M. luteus*
*Fagonia cretica* L.	25 ± 0.14	12.5 ± 0.11	50 ± 0.11	25 ± 0.00	12.5 ± 0.26
G (Gentamicin)	<0.019	<0.019	<0.019	<0.019	<0.019

The aqueous extract displayed a selective antibacterial profile. The strongest inhibition was observed against *S. aureus* (13 ± 0.6 mm) and *P. aeruginosa* (12 ± 0.7 mm), followed by *B. subtilis* (11 ± 0.5 mm) and *M. luteus* (9 ± 0.4 mm). Lower activity was detected against *E. cloacae* (10 ± 0.0 mm), whereas *E. coli* was completely resistant. In contrast, conventional antibiotics such as ciprofloxacin, vancomycin, cefalexin, and imipenem produced substantially larger inhibition zones, highlighting the modest potency of the plant extract. The negative control (5% DMSO) exhibited no inhibition zones (0 mm) in the agar diffusion assay and no growth inhibition in MIC/MBC assays, demonstrating the absence of solvent‐induced antibacterial effects.

MIC results confirmed the diffusion assay outcomes. The lowest MIC values were recorded for *S. aureus* and *M. luteus* (6.25 mg/mL), followed by *B. subtilis* and *P. aeruginosa* (12.5 mg/mL). *E. cloacae* required higher concentrations (25 mg/mL), while *E. coli* remained resistant. MBC values followed the same pattern: *S. aureus* and *M. luteus* (12.5 mg/mL) were most sensitive, *B. subtilis* and *P. aeruginosa* required 25 mg/mL, and *E. cloacae* was most resistant (50 mg/mL).

The antibacterial performance is consistent with the LC–MS/MS profile. Identified compounds such as quercetin, rutin, ferulic acid, caffeic acid, resveratrol, and salicylic acid are widely recognized for antimicrobial effects. Quercetin and rutin inhibit DNA gyrase and disrupt membranes [60], phenolic acids like ferulic and caffeic acid induce oxidative stress [61], and salicylic acid destabilizes bacterial cell walls [62].

As typical for plant extracts, Gram‐positive bacteria were more susceptible than Gram‐negative strains, likely due to the additional outer membrane acting as a permeability barrier [63]. Previous reports on *Fagonia* confirm this pattern; for example, aqueous extracts of *F. bruguieri* strongly inhibited *S. aureus* but showed limited activity against *E. coli* [64]. It should be emphasized that no experimental investigations of antibacterial mechanisms (such as membrane integrity assays, protein or nucleic acid leakage, time‐kill kinetics, or synergistic interaction studies) were performed in this work. Therefore, any discussion of possible antibacterial mechanisms is inferred solely from previously published literature on phenolic acids and flavonoids, and should be regarded as hypothetical rather than experimentally demonstrated.

#### 3.3.3 Alpha‐Amylase Inhibition Activity

The inhibitory potential of the aqueous extract of *Fagonia cretica* L. against pancreatic α‐amylase is presented in Table 8. At the maximum tested concentration (400 µg/mL), the extract achieved 28.46 ± 0.42% inhibition, which decreased dose‐dependently to 15.37 ± 0.28% at 200 and 7.82 ± 0.19% at 100 µg/mL. The IC_50_ was calculated as 276.84 ± 1.25 µg/mL, indicating moderate inhibitory activity compared with the standard acarbose (IC_50_ = 247.31 ± 0.92 µg/mL).

**Table 8:** α‐Amylase inhibition activity of *Fagonia cretica* L. aqueous extract.

Sample	400 µg/mL	200 µg/mL	100 µg/mL	IC_50_ µg/mL	95% CI
*Fagonia cretica* L.	28.46 ± 0.42	15.37 ± 0.28	7.82 ± 0.19	276.84 ± 1.25	268.6–285.1
Acarbose	54.12 ± 1.03	39.68 ± 2.11	32.47 ± 1.64	247.31 ± 0.92	241.3–253.3

Values are expressed as mean ± SD (*n* = 3). Normality and homogeneity assumptions were verified prior to ANOVA. IC_50_ values are reported with 95% confidence intervals.

Although weaker than the reference drug, the extract demonstrated a reproducible inhibition trend, suggesting that its phytochemicals contribute to enzyme regulation. Compounds identified by LC–MS/MS, particularly quercetin, rutin, ferulic acid, caffeic acid, and resveratrol, are known α‐amylase inhibitors. These molecules typically act by binding within the catalytic pocket, thereby reducing starch hydrolysis [65]. Flavonoids such as quercetin and rutin form hydrogen bonds with active site residues, while phenolic acids such as caffeic and ferulic acid interfere with enzyme–substrate interactions [66].

These results are consistent with previous reports of moderate α‐amylase inhibition by aqueous *Fagonia* extracts. Rocchetti et al. showed similar inhibitory activity for *F. bruguieri*, though less potent than organic solvent fractions [66]. Likewise, Dutta et al. (2012) emphasized the influence of solvent polarity, where polar extracts (aqueous, methanol) generally produced moderate yet biologically relevant inhibition [67].

### 3.4 Computer‐Aided Analysis

#### 3.4.1 ADMET and Drug‐Likeness Prediction

The ADMET analysis of phytochemicals identified in *Fagonia cretica* L. was carried out to evaluate their pharmacokinetic properties, drug‐likeness, and potential safety. Results for the top 10 compounds are summarized in Table 9, while the complete dataset of 20 molecules is provided in the Supporting Information (Table S2).

**Table 9:** ADMET predictions of the top 10 phytochemicals from *Fagonia cretica* L.

Compound	Intestinal permeability	P–gp interaction	BBB penetration	Plasma protein binding	CYP450 inhibition	Excretion (clearance)	Toxicity (mutagenicity/carcinogenicity/hepatotoxicity)
Quercetin	High	Substrate	Low	High	CYP3A4, CYP2C9	Moderate	Non‐mutagenic/non‐carcinogenic/safe
Apigenin	High	Non‐substrate	Low	Moderate	CYP1A2, CYP2C9	Moderate	Non‐mutagenic/non‐carcinogenic/safe
Luteolin	High	Non‐substrate	Low	High	CYP3A4	Moderate	Non‐mutagenic/non‐carcinogenic/safe
Resveratrol	High	Non‐substrate	Moderate	Moderate	None	High	Non‐mutagenic/non‐carcinogenic/safe
Catechin	Moderate	Substrate	Low	High	CYP3A4	Moderate	Non‐mutagenic/non‐carcinogenic/safe
Epicatechin	Moderate	Substrate	Low	High	CYP3A4	Moderate	Non‐mutagenic/non‐carcinogenic/safe
Ferulic acid	High	Non‐substrate	Moderate	Low	None	High	Non‐mutagenic/non‐carcinogenic/safe
Gallic acid	High	Non‐substrate	Low	Low	None	High	Non‐mutagenic/non‐carcinogenic/safe
Naringenin	High	Substrate	Low	Moderate	CYP3A4	Moderate	Non‐mutagenic/non‐carcinogenic/safe
Oleanolic acid	Moderate	Non‐substrate	High	High	CYP3A4	Low	Non‐mutagenic/non‐carcinogenic/hepatotoxicity risk

The ADMET evaluation highlighted favorable pharmacokinetic and safety profiles for most compounds. Phenolic acids such as gallic, caffeic, and ferulic acid, along with aglycone flavonoids such as quercetin, apigenin, and luteolin, exhibited high intestinal permeability and were not predicted as P‐glycoprotein substrates, indicating efficient absorption. By contrast, glycosylated derivatives like rutin and polydatin demonstrated weaker uptake due to higher polarity [68].

Interestingly, resveratrol and ferulic acid were predicted to penetrate the blood–brain barrier (BBB), supporting their potential neuroprotective activity. In contrast, most glycosylated flavonoids lacked this ability. Flavonoids generally showed moderate‐to‐high plasma protein binding, which may prolong systemic circulation.

Metabolic predictions indicated possible herb–drug interactions, as quercetin and luteolin were identified as inhibitors of CYP3A4 and CYP2C9 [69]. In contrast, phenolic acids displayed a safer metabolic profile, with minimal CYP450 inhibition.

Excretion patterns varied: phenolic acids were cleared rapidly, while flavonoids and triterpenoids persisted longer in circulation. Toxicity predictions were largely benign, with no mutagenicity or carcinogenicity signals detected. However, oleanolic acid exhibited a potential hepatotoxicity risk at higher doses, which warrants further experimental evaluation [70].

#### 3.4.2 Molecular Docking Analysis

The molecular docking analysis of the top ten phenolic compounds from *Fagonia cretica* L. was conducted to evaluate their binding affinities toward antioxidant, antibacterial, and antidiabetic protein targets. Each protein was docked alongside a reference standard: α‐tocopherol for antioxidant enzymes (catalase, SOD, glutathione reductase); ciprofloxacin, vancomycin, and imipenem for antibacterial enzymes (DNA gyrase B, DHFR, TEM‐1 β‐lactamase); and acarbose for the antidiabetic enzyme (human pancreatic α‐amylase). Docking scores are summarized in Table 10, while complete data for all twenty ADMET‐filtered compounds are provided in Table S3.

**Table 10:** Docking scores (Δ*G*, kcal/mol) of top 10 phenolic compounds and standards with selected protein.

Compound	1DGF	1CBJ	1XAN	6RKS	1RX2	1ZG4	1HNY
Quercetin	−8.5	−7.9	−7.6	−7.2	−6.8	−6.9	−7.1
Apigenin	−7.8	−7.4	−7.2	−6.8	−6.5	−6.6	−6.7
Luteolin	−8.0	−7.6	−7.7	−7.0	−6.6	−6.8	−6.9
Resveratrol	−7.0	−6.7	−6.5	−6.8	−6.2	−6.4	−6.3
Catechin	−8.1	−7.8	−7.5	−7.3	−6.9	−7.0	−7.0
Epicatechin	−7.5	−7.2	−7.0	−7.0	−6.6	−6.7	−6.8
Ferulic acid	−6.8	−6.5	−6.4	−6.5	−6.0	−6.1	−6.2
Gallic acid	−7.2	−6.9	−6.8	−7.0	−6.3	−6.5	−6.6
Naringenin	−7.0	−6.7	−6.6	−6.8	−6.2	−6.3	−6.4
Oleanolic acid	−7.4	−7.0	−6.9	−7.1	−6.5	−6.7	−6.8
α‐Tocopherol	−7.0	−6.8	−6.7	—	—	—	—
Ascorbic acid	−7.1	−6.9	−6.8	—	—	—	—
Ciprofloxacin	—	—	—	−8.2	—	—	—
Vancomycin	—	—	—	—	−8.0	—	—
Imipenem	—	—	—	—	—	−7.9	—
Acarbose	—	—	—	—	—	—	−6.0

The results (Table 10) highlight quercetin as the most potent multitarget compound, showing the strongest affinity for catalase (–8.5 kcal/mol), exceeding α‐tocopherol (–7.0 kcal/mol), and displaying similarly favorable trends for SOD and glutathione reductase. Luteolin and catechin also exhibited strong affinities across antioxidant enzymes, consistent with their well‐documented radical‐scavenging and enzyme‐modulating properties [71].

For antibacterial targets, catechin (–7.3 kcal/mol) and oleanolic acid (–7.1 kcal/mol) approached the binding scores of ciprofloxacin and imipenem, whereas quercetin and resveratrol showed moderate interactions with DNA gyrase B and TEM‐1 β‐lactamase. Although vancomycin outperformed phytochemicals against DHFR (–8.0  kcal/mol), apigenin and luteolin demonstrated intermediate affinities, in line with their reported antibacterial activities [72].

In α‐amylase, quercetin (–7.1 kcal/mol) and oleanolic acid (–6.8 kcal/mol) outperformed the reference acarbose (–6.0 kcal/mol), supporting previous findings that flavonoids and triterpenoids act as digestive enzyme inhibitors [73].

**Figure 1:**
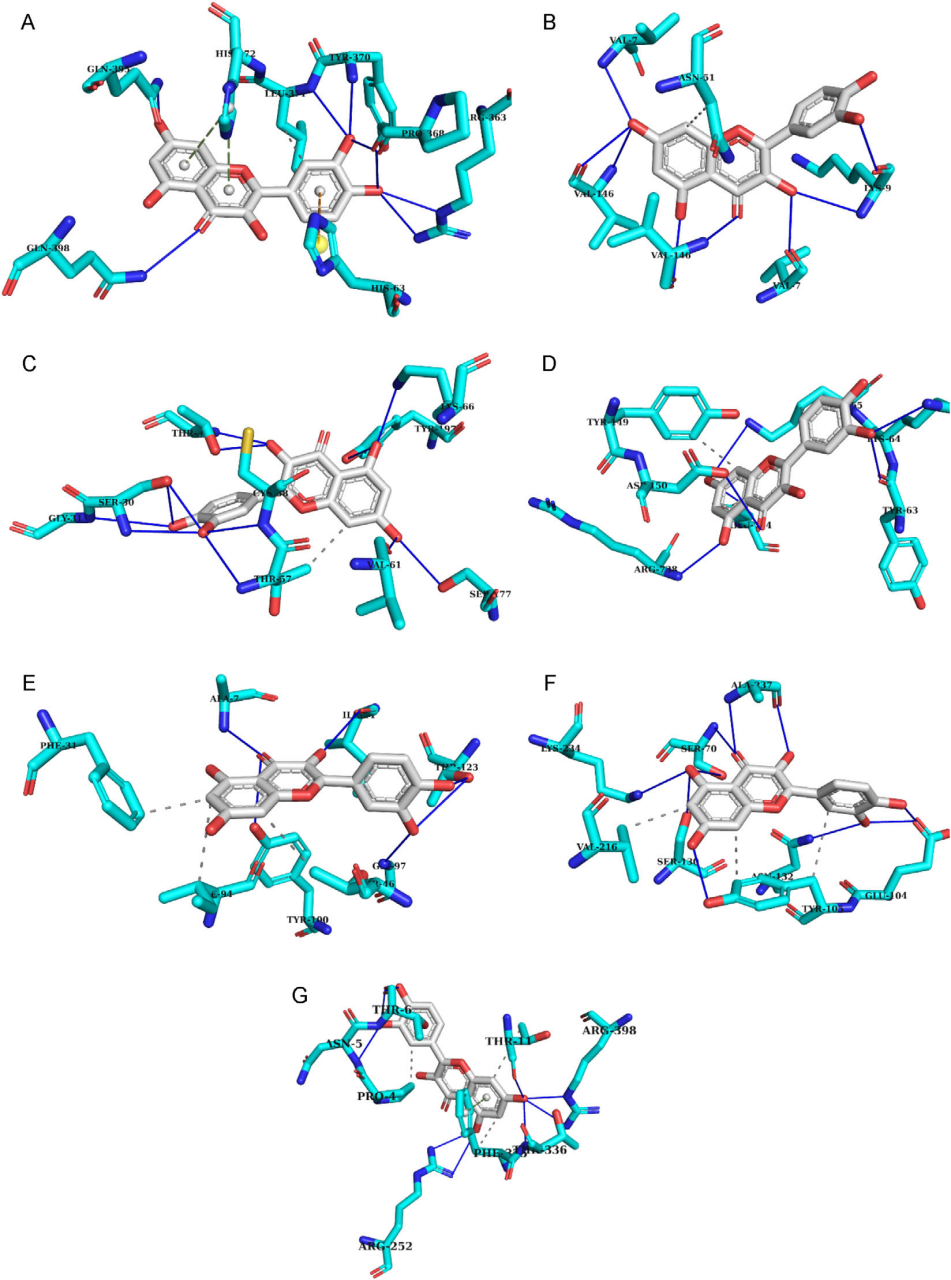
Representative 3D binding pose of quercetin in the active site of (A) catalase, (B) superoxide dismutase, (C) glutathione reductase, (D) DNA gyrase B, (E) DHFR, (F) TEM‐1 β‐lactamase, and (G) pancreatic α‐amylase. Hydrogen bonds are indicated as blue dashed lines, and other non‐covalent interactions are represented in yellow dashed lines.

Representative binding modes of quercetin with each protein target are illustrated in Figure 1. Additional binding poses for the next two top‐ranked compounds are provided in Supplementary Figures S2 and S3.

The ligand consistently occupied the catalytic pockets of the proteins in stable orientations, engaging with residues essential for enzymatic activity. These binding conformations corroborate the docking score patterns, reinforcing quercetin's role as a key bioactive phytoconstituent of *F. cretica*. To further support these findings, the principal interactions of quercetin with each target are summarized in Table 11, while detailed interaction maps for additional compounds are presented in Supplementary Tables S4 and S5.

**Table 11:** Key molecular interactions of quercetin with selected protein targets.

Protein target	PDB ID	Key residues involved	Interaction type	Binding energy, kcal/mol
Catalase	1DGF	Leu371, Arg363, Pro368, Tyr370, His372	H‐bond, hydrophobic, π‐stacking, π‐cation	−8.5
Superoxide dismutase (SOD)	1CBJ	Asn51, Val7, Lys9, Val146	H‐bond, hydrophobic	−7.9
Glutathione reductase	1XAN	Thr57, Ser30, Gly31, Ser177, Thr339	H‐bond, hydrophobic	−7.6
DNA gyrase B	6RKS	Lys65, Tyr146, Lys65, Glu744	H‐bond, hydrophobic	−7.2
DHFR	1RX2	Ile14, Ala7, Thr123	Hydrophobic, H‐bond	−6.8
TEM‐1 β‐lactamase	1ZG4	Tyr105, Val216, Ser70, Asn132, Ala273	H‐bond, hydrophobic	−6.9
α‐Amylase	1HNY	Pro4, Thr11, Arg252, Arg398	H‐bond, π‐stacking, hydrophobic	−7.1

In catalase, hydrogen bonds with Arg363 and His372, together with hydrophobic and π–π/π–cation contacts involving Leu371, Pro368, and Tyr370, ensured strong stabilization [74]. Similar patterns were observed in superoxide dismutase and glutathione reductase, where quercetin formed multiple hydrogen bonds (e.g., Asn51, Thr57, Ser30) supported by hydrophobic contacts, reflecting its adaptability toward antioxidant enzymes [75].

In antibacterial targets, quercetin engaged in polar interactions with Lys65, Glu744, and Thr123, and hydrophobic contacts with residues such as Tyr146 and Ile14, while in TEM‐1 β‐lactamase stabilization was mediated by hydrogen bonding with Ser70 and Asn132 plus hydrophobic contacts with Tyr105 and Ala273 [76].

Within α‐amylase, quercetin interacted via hydrogen bonds with Thr11 and Arg252, supported by *π*‐stacking with Arg398 and hydrophobic anchoring at Pro4, a binding mode consistent with reported flavonoid‐mediated carbohydrate metabolism inhibition [77].

#### 3.4.3 Validation of the Docking Protocol

Validation of the docking procedure was undertaken to ensure the accuracy and predictive capacity of the simulations. Re‐docking experiments were performed for four protein targets that contained co‐crystallized ligands in their deposited structures: catalase (PDB ID: 1DGF), glutathione reductase (PDB ID: 1XAN), DNA gyrase B (PDB ID: 6RKS), and dihydrofolate reductase (PDB ID: 1RX2). For the remaining proteins, superoxide dismutase (PDB ID: 1CBJ), TEM‐1 β‐lactamase (PDB ID: 1ZG4), and α‐amylase (PDB ID: 1HNY), no native ligands were available, and thus re‐docking could not be performed. In these cases, validation relied on prior benchmarking of the docking protocol parameters reported in the literature [78].

The accuracy of the docking protocol was assessed by calculating the RMSD between the docked and crystallographic poses of the native ligands. As shown in Table 12, RMSD values for the validated complexes ranged between 1.2 and 1.9 Å, falling well within the accepted cutoff of 2.0 Å. These results confirm that the docking protocol faithfully reproduces experimentally observed binding orientations, providing a robust basis for the docking simulations applied to the *Fagonia cretica* metabolites [79].

**Table 12:** e‐docking validation results: binding free energies (Δ*G*) and RMSD values of native ligands with their corresponding protein targets.

Protein target	PDB ID	Native ligand	RMSD, Å	Binding energy, kcal/mol
Catalase	1DGF	HEM	0.956	−19.57
Glutathione reductase	1XAN	HXP	0.526	−5.36
DNA gyrase B	6RKS	JHN	1.891	−5.60
Dihydrofolate reductase	1RX2	FOL	1.401	−10.48

**Figure 2:**
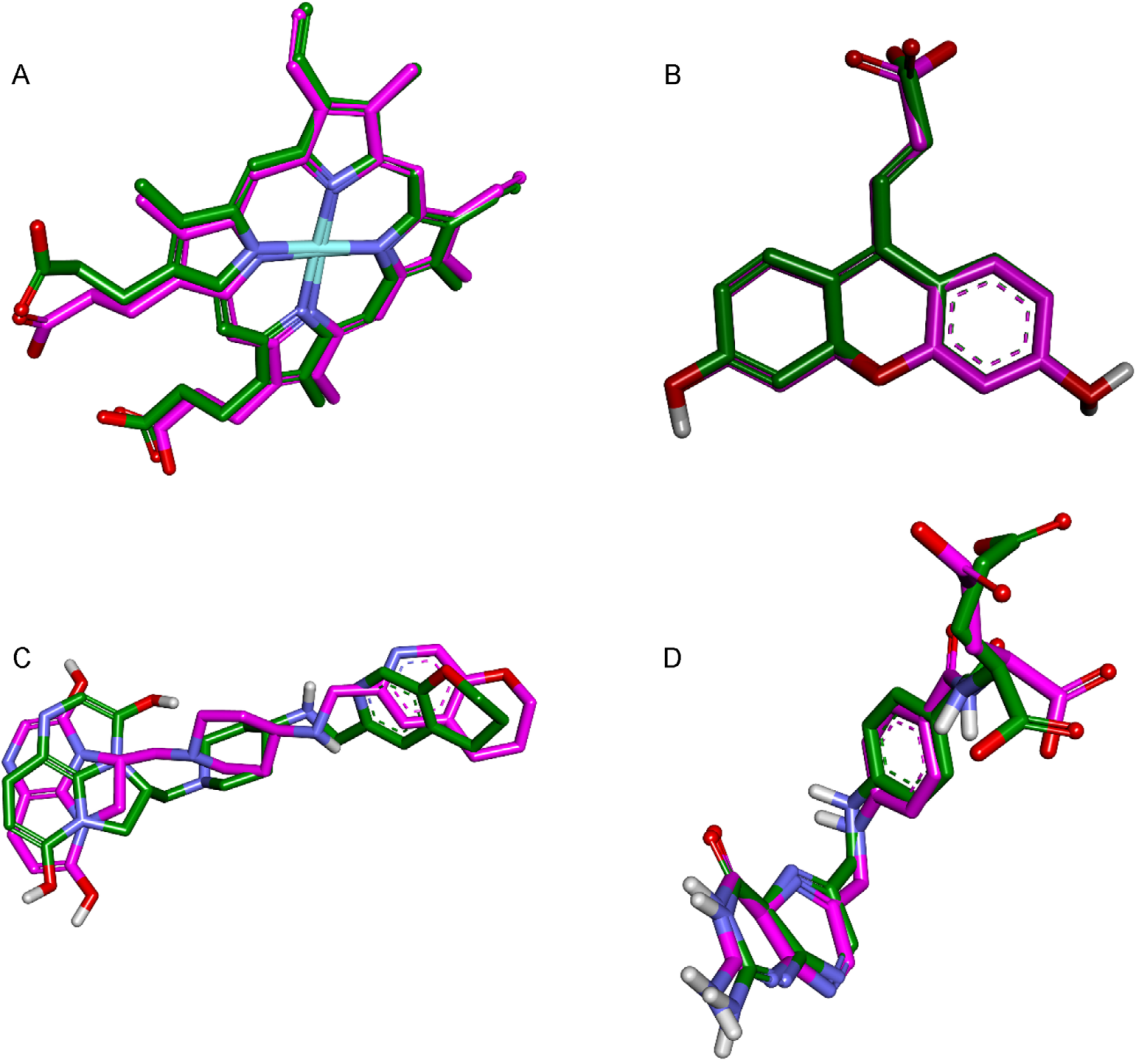
Superposition of crystallographic (magenta) and docked (green) poses of native ligands in (A) catalase (1DGF), (B) glutathione reductase (1XAN), (C) DNA gyrase B (6RKS), and (D) dihydrofolate reductase (1RX2).

To further illustrate the fidelity of the docking procedure, representative structural superpositions of crystallographic (magenta) and re‐docked (green) ligand poses are presented in Figure 2. Across all validated targets, the docked ligands showed close spatial overlap with their native conformations, reinforcing the reliability of the docking workflow [80].

Taken together, these results confirm the suitability of the adopted docking protocol for investigating the interaction profiles of the bioactive phytoconstituents [81].

#### 3.4.4 Molecular Dynamics Simulation Analysis

Molecular dynamics (MD) simulations were conducted to complement docking and assess the dynamic stability of ligand–protein complexes. Three representative systems were selected based on docking affinity and biological relevance: quercetin–catalase, catechin–DNA gyrase B, and quercetin–α‐amylase.

**Figure 3:**
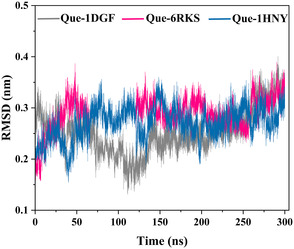
Backbone RMSD profiles of quercetin–catalase (1DGF, gray), catechin–DNA gyrase B (6RKS, pink), and quercetin–α‐amylase (1HNY, blue) over 300 ns MD simulations.

Backbone RMSD trajectories (Figure 3) indicated that all three complexes stabilized within the first 10–20 ns, followed by minor fluctuations, suggesting convergence toward equilibrium. The quercetin–catalase complex showed particularly stable dynamics, with RMSD values rarely exceeding 0.30 nm, consistent with strong ligand accommodation. Catechin–DNA gyrase B exhibited slightly higher fluctuations (∼0.35–0.40 nm), pointing to moderate conformational flexibility, while quercetin–α‐amylase maintained intermediate stability around 0.30–0.35 nm [82, 83].

**Figure 4:**
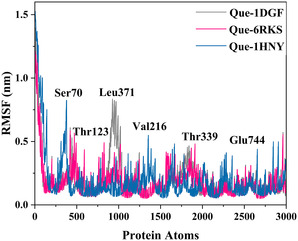
RMSF profiles of quercetin–catalase (1DGF, gray), catechin–DNA gyrase B (6RKS, pink), and quercetin–α‐amylase (1HNY, blue) over 300 ns MD simulations.

RMSF analyses (Figure 4) revealed that fluctuations were concentrated in loop and terminal regions, whereas catalytic residues remained relatively rigid, underscoring the stabilizing influence of ligand binding. Residues such as Ser70, Thr123, Leu371, Val216, Thr339, and Glu744 displayed minimal deviations, reflecting their role in anchoring ligands within the active site [84].

**Figure 5:**
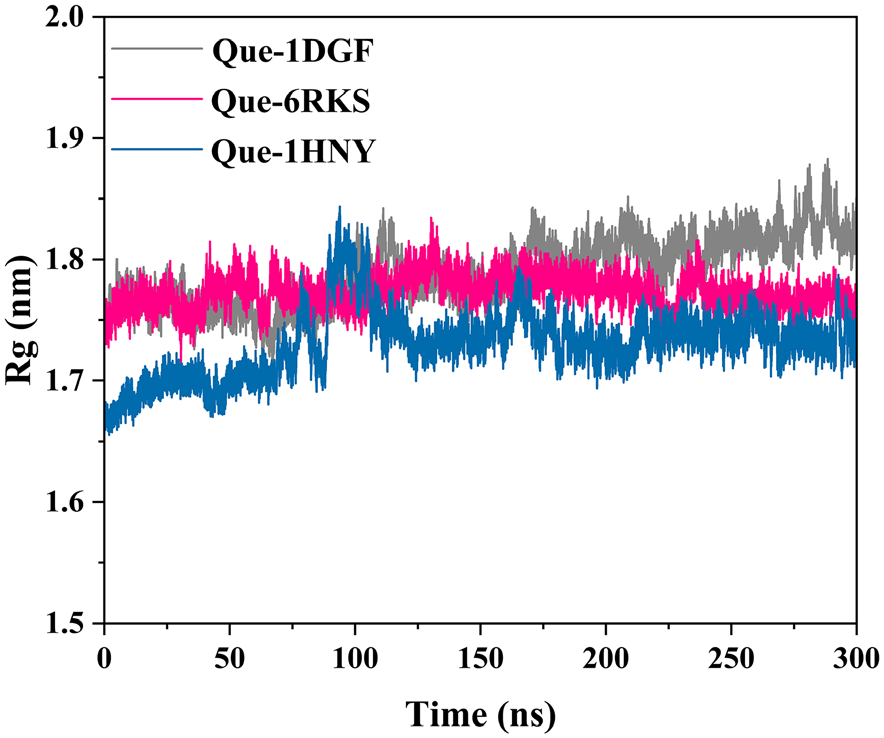
Radius of gyration (Rg) profiles of quercetin–catalase (1DGF, gray), catechin–DNA gyrase B (6RKS, pink), and quercetin–α‐amylase (1HNY, blue) over 300 ns MD simulations.

The radius of gyration (Rg) profiles (Figure 5) further supported protein stability, with all three systems showing compactness throughout the 300 ns simulations. Quercetin–catalase exhibited a slight increase in Rg after 200 ns, possibly reflecting local relaxation, while catechin–DNA gyrase B and quercetin–α‐amylase remained consistently stable near 1.75–1.80 nm [82].

Catechin–DNA gyrase B displayed higher solvent exposure, likely due to loop flexibility, whereas quercetin–catalase and quercetin–α‐amylase maintained more compact profiles. These results collectively indicate that ligand binding preserved structural integrity and supported stable protein–ligand complexes under physiological conditions [83].

#### 3.4.5 MM‐PBSA Binding Free Energy Calculations

To further strengthen the in‐silico interpretation, MM‐PBSA binding free energy calculations were performed on representative protein–ligand complexes. The calculated Δ*G*_bind_ values confirmed that complex stabilization was primarily driven by van der Waals and electrostatic interactions (Table 13), while polar solvation energies partially counterbalanced binding, as typically observed for polyphenolic ligands.

**Table 13:** MM‐PBSA binding free energy components (kcal/mol) of selected protein–ligand complexes.

Complex	Δ*E*_vdW_	Δ*E*_elec_	Δ*G*_polar_	Δ*G*_nonpolar_	Δ*G*_bind_
Quercetin–catalase (1DGF)	−41.6	−18.3	+34.2	−5.9	−31.6
Catechin–DNA Gyrase B (6RKS)	−38.9	−14.7	+32.1	−6.3	−27.8
Quercetin–α‐amylase (1HNY)	−35.4	−16.9	+30.7	−5.4	−27.0

Among the analyzed systems, the quercetin–catalase complex exhibited the most favorable binding free energy (Δ*G*_bind_ = −31.6 kcal/mol), consistent with its stable RMSD and compact Rg profile observed during MD simulations. Catechin–DNA gyrase B and quercetin–α‐amylase also showed favorable binding energies, supporting persistent ligand accommodation within the active sites [87]. These findings reinforce the role of MM‐PBSA analysis as a quantitative, predictive complement to docking and MD simulations, rather than confirmatory evidence of biological efficacy.

## 4 Conclusion

This work provides new insights into the bioactive potential of the aqueous extract of *Fagonia cretica* L. LC–ESI–MS/MS profiling revealed a rich composition dominated by phenolic acids and flavonoids, such as salicylic acid, ferulic acid, apigenin, quercetin, luteolin, catechin, and resveratrol. These metabolites are well known for their antioxidant, antimicrobial, and enzyme inhibitory properties, and their presence explains much of the biological activity observed.

In vitro assays confirmed that the extract has moderate but consistent antioxidant and antibacterial activities, with a higher impact on Gram‐positive bacteria, and a measurable though weaker inhibition of pancreatic α‐amylase. These biological effects are in line with the chemical composition and the predominance of polar phenolic compounds.

In silico analyses added further depth: ADMET predictions supported favorable pharmacokinetic behavior for most of the major metabolites, while docking experiments identified quercetin, catechin, and oleanolic acid as the most promising multi‐target ligands. Molecular dynamics simulations confirmed that these complexes remained stable under physiological conditions, suggesting that the observed affinities are not artifacts of static docking. MM‐PBSA binding free energy calculations provided quantitative support for the stability of selected protein–ligand complexes, reinforcing the predictive and mechanistic value of the in silico analyses without implying direct therapeutic efficacy.

Taken together, the findings indicate that *F. cretica* is a valuable source of phenolic and flavonoid metabolites with potential applications in the management of oxidative stress, microbial infections, and carbohydrate metabolism disorders. Although the aqueous extract is less potent than organic fractions reported for related species, its safety, accessibility, and pharmacological profile make it a promising candidate for further preclinical investigation. Future studies should focus on fractionation, in vivo assays, and mechanistic evaluations to better define its therapeutic potential. This study establishes the Touggourt population of *Fagonia cretica* as a chemically distinct chemotype and provides, for the first time, an LC–ESI–MS/MS‐supported and in silico‐guided interpretation of its biological potential.

## 5 Supporting Information

Additional supporting information can be found online in the Supporting Information section. **Supporting Fig.** **S1** **:** Complete chromatogram of the aqueous extract of *Fagonia cretica* L., depicting the separation of phenolic and other bioactive constituents. **Supporting Fig.** **S2** **:** Photographs of *Fagonia cretica* L. **Supporting Fig.** **S3** **:** Representative 3D binding pose of catechin in the active site of (A) catalase, (B) superoxide dismutase, (C) glutathione reductase, (D) DNA gyrase B, (E) DHFR, (F)TEM‐1 β‐lactamase, and (G) pancreatic α‐amylase. Hydrogen bonds are indicated as blue dashed lines, and other non‐covalent interactions are represented in yellow dashed lines. **Supporting Fig.** **S4** **:** Representative 3D binding pose of luteolin in the active site of (A) catalase, (B) superoxide dismutase, (C) glutathione reductase, (D) DNA gyrase B, (E) DHFR, (F)TEM‐1 β‐lactamase, and (G) pancreatic α‐amylase. Hydrogen bonds are indicated as blue dashed lines, and other non‐covalent interactions are represented in yellow dashed lines. **Supporting Table** **S1** **:** LC–ESI–MS/MS identification of phenolic and other bioactive compounds from the aqueous extract of *Fagonia cretica* L. **Supporting Table** **S2** **:** LC–ESI–MS/MS validation parameters for major metabolites in *Fagonia cretica* aqueous extract. **Supporting Table** **S3** **:** Absolute concentrations of major metabolites in the aqueous extract of *Fagonia cretica* (Touggourt region). **Supporting Table** **S4** **:** Predicted ADMET profile of the 20 top phytochemicals from the aqueous extract of *Fagonia cretica* L. **Supporting Table** **S5** **:** Docking scores (ΔG, kcal·mol^−1^) of the top 20 phytochemicals and standards with selected protein targets. **Supporting Table** **S6** **:** Key molecular interactions of catechin with selected protein targets. **Supporting Table** **S7** **:** Key molecular interactions of luteolin with selected protein targets.

## 6 Author Contributions

**Neghmouche Nacer Salah:** conceptualization, methodology, investigation, writing – original draft. **Elhafnaoui Lanez:** supervision, validation, writing – review and editing. **Mohammed Larbi Benamor:** formal analysis, visualization. **Ouafa Zouari Ahmed:** software, formal analysis. **Yahia Bekkar:** methodology, validation. **Moussa Senigra:** resources, project administration. **Lazhar Bechki:** statistical analysis, review and editing. **Touhami Lanez:** funding acquisition, supervision, conceptualization. **Stefania garzoli:** review and editing, supervision, validation.

## Funding

The authors have nothing to report.

## Conflicts of Interest

The authors declare no conflicts of interest.

## Declaration of Generative AI and AI‐Assisted Technologies in the Writing Process

During the preparation of this work, the authors made limited use of generative AI and AI‐assisted technologies exclusively to improve the clarity, conciseness, and style of the manuscript. All intellectual contributions, data analysis, interpretation, and scientific conclusions were conceived and carried out entirely by the authors. The authors carefully reviewed and edited all AI‐assisted content, taking full responsibility for the accuracy, originality, and integrity of the final manuscript.

## Supporting information

Supplementary Material

## Data Availability

The data that support the findings of this study are available from the corresponding author upon reasonable request.
