# Supplementary material for: Liquid Chromatography‐Electrospray Ionization‐Tandem Mass Spectrometry Profiling, Antioxidant, Antibacterial, and Antidiabetic Properties of Fagonia cretica L.: Insights From In Vitro and In Silico Approaches
Source: ChemistryOpen. 2026 May 21;15(6):e202500592. doi: 10.1002/open.202500592 (PMC13240333; doi:10.1002/open.202500592)
Supplement: Supplementary file 1 — Supplementary Material [file OPEN-15-e202500592-s001.pdf]

LC–ESI–MS/MS Profiling, Antioxidant, Antibacterial, and Antidiabetic Properties of *Fagonia cretica L.*: Insights from *In Vitro* and *In Silico* Approaches

Neghmouche Nacer Salah[1], Elhafnaoui Lanez[1], Mohammed Larbi Benamor[1], Ouafa Zouari Ahmed[2], Yahia Bekkar[1], Moussa Senigra[3,4], Lazhar Bechki[5,6], Touhami Lanez[1]

[1] University of El Oued, Faculty of exact Sciences, Department of Chemistry, VTRS Laboratory, B.P.789, 39000, El Oued, Algeria

[2] Laboratory of Biology, Environment, and Health, Faculty of Natural and Life Sciences, University of El-Oued, El-Oued 39000, Algeria

[3] Laboratory of Applied Chemistry and Environment (LCAE), Chemistry Department, University of Hamma Lakhdar El Oued, B.P. 789, El Oued 39000, Algeria

[4] University of El Oued, Faculty of Natural Sciences and Life, Department of Biology, 39000, El Oued, Algeria

[5] Kasdi Merbah University, VPRS Laboratory, Department of Chemistry, Faculty of Mathematics and Material Sciences, 30000 Ouargla, Algeria

[6] Kasdi Merbah University, Faculty of Mathematics and Material Sciences, Department of Chemistry, 30000, Ouargla, Algeria

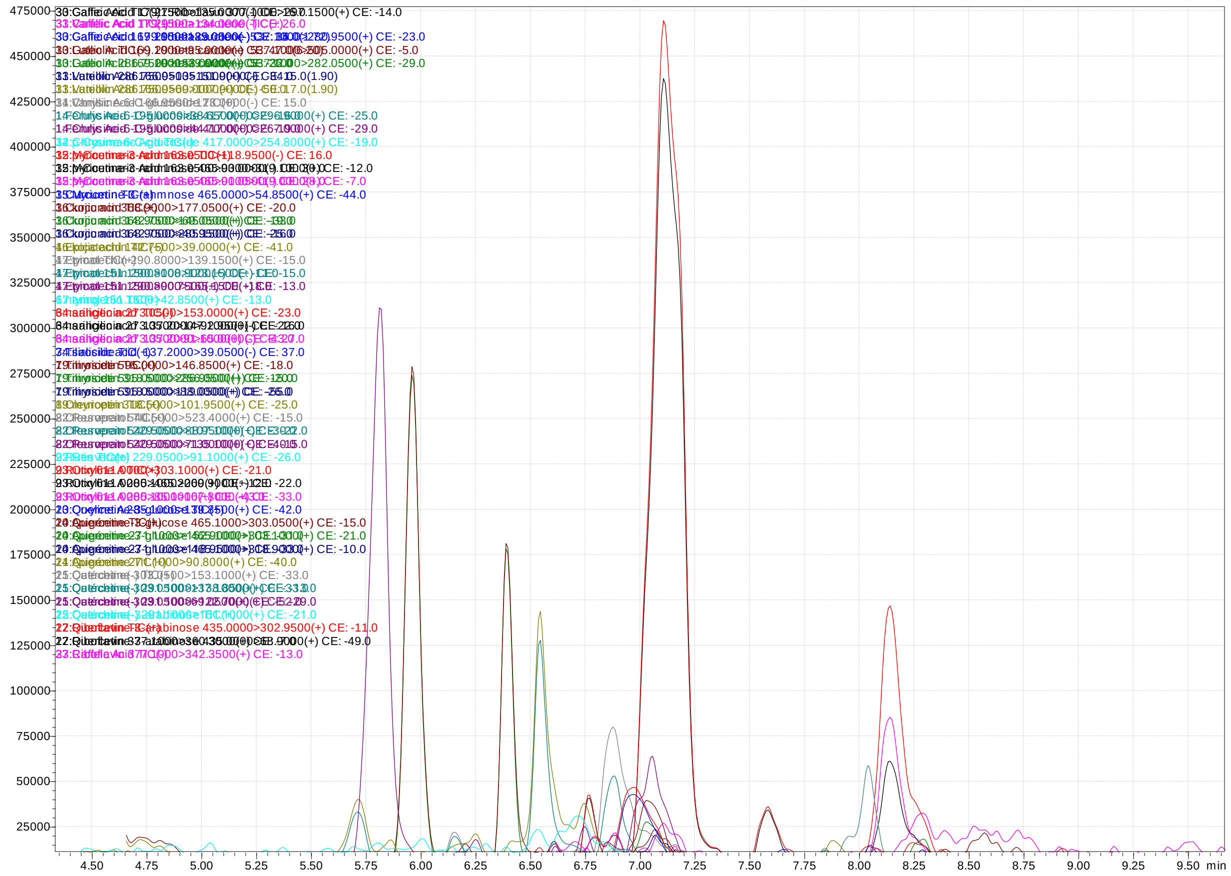

Figure S1. Complete chromatogram of the aqueous extract of *Fagonia cretica* L., depicting the separation of phenolic and other bioactive constituents.

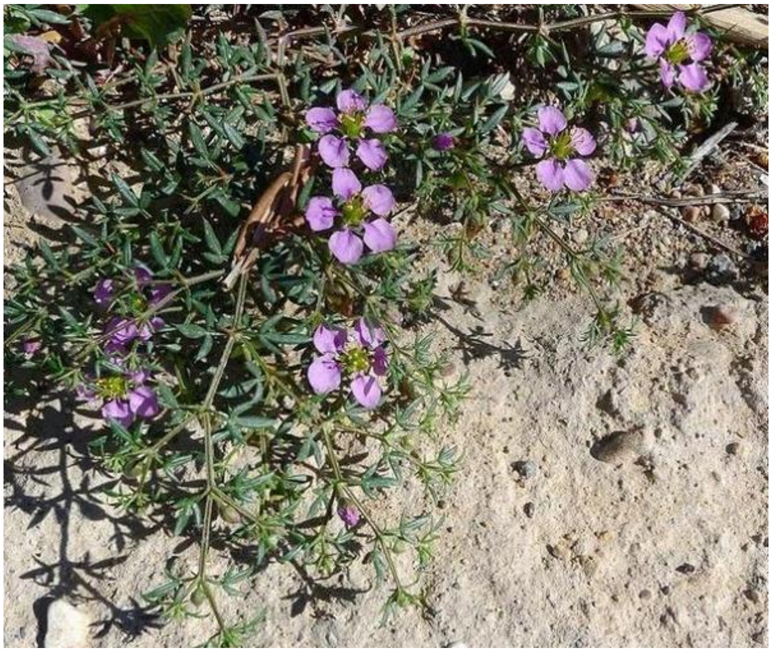

Figure S2. Photographs of *Fagonia cretica* L.

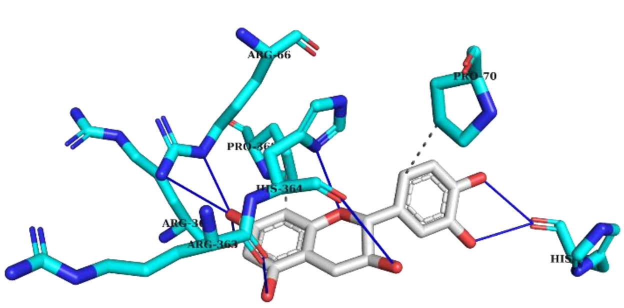

A

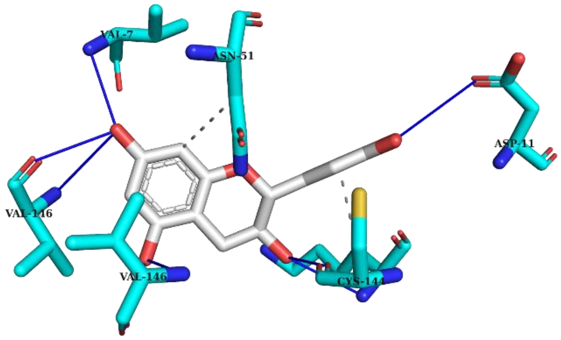

B

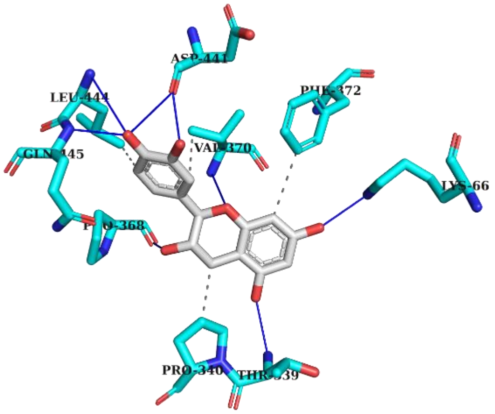

C

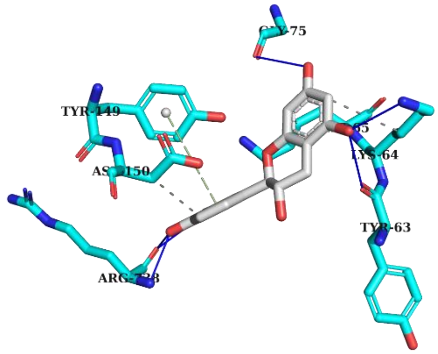

D

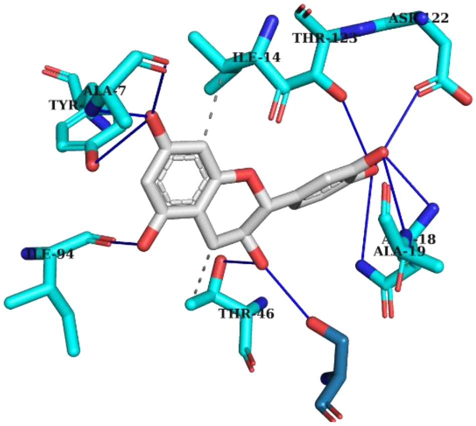

E

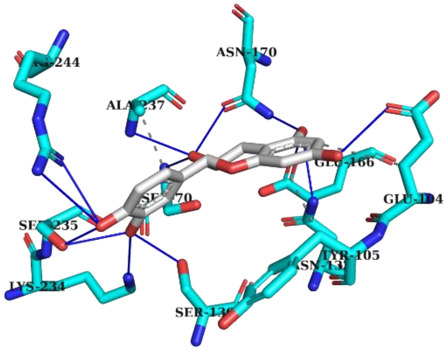

F

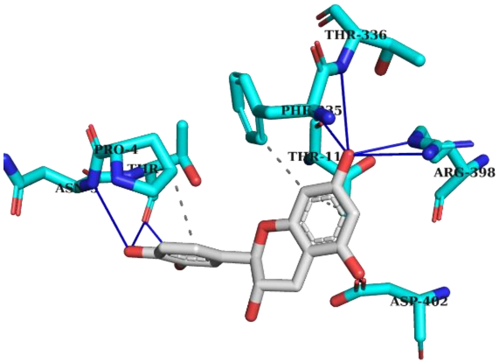

G

Figure S3. Representative 3D binding pose of catechin in the active site of (A) catalase, (B) superoxide dismutase, (C) glutathione reductase, (D) DNA gyrase B, (E) DHFR, (F)TEM-1 β-lactamase, and (G) pancreatic α-amylase. Hydrogen bonds are indicated as blue dashed lines, and other non-covalent interactions are represented in yellow dashed lines.

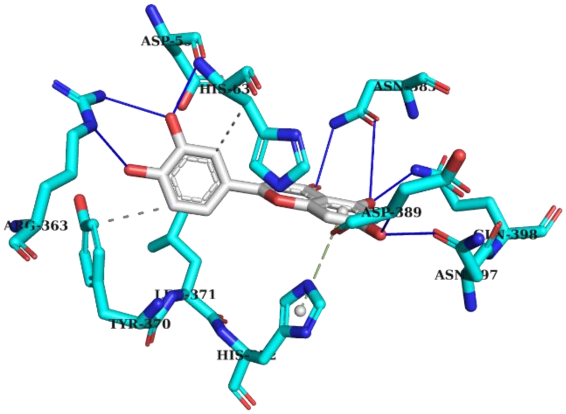

A

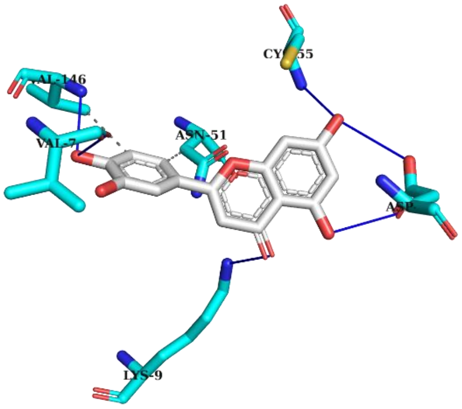

B

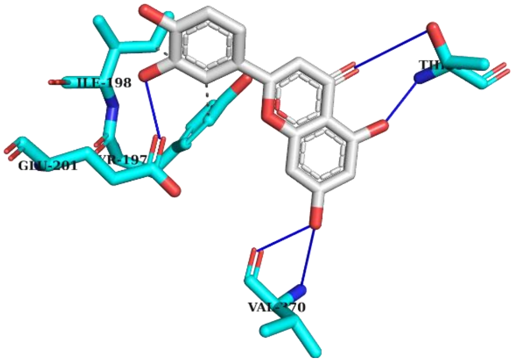

C

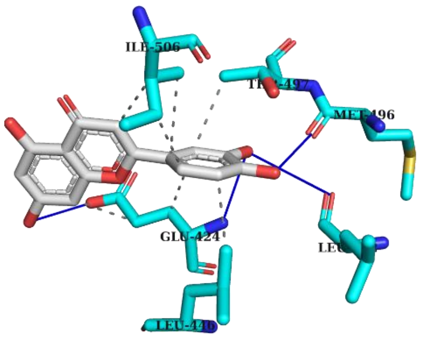

D

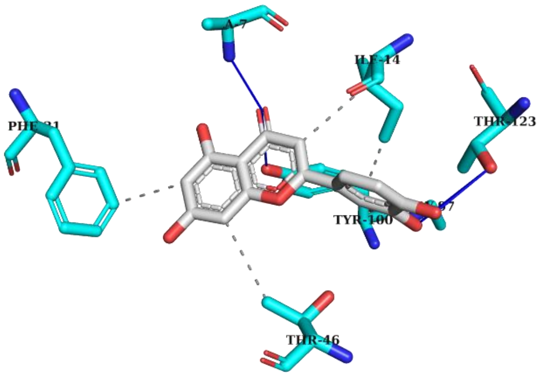

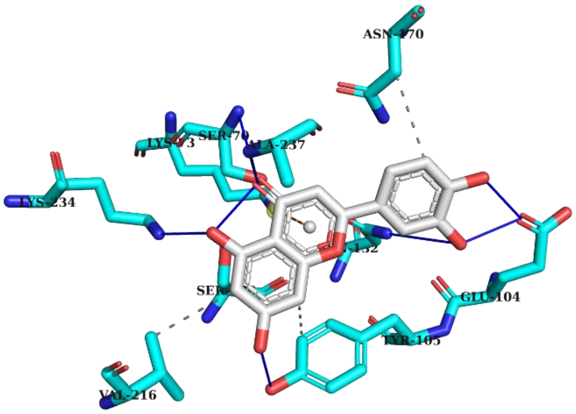

E F

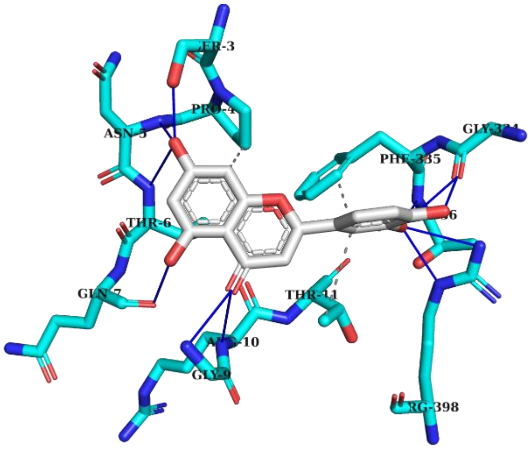

G

Figure S4. Representative 3D binding pose of luteolin in the active site of (A) catalase, (B) superoxide dismutase, (C) glutathione reductase, (D) DNA gyrase B, (E) DHFR, (F)TEM-1 β-lactamase, and (G) pancreatic α-amylase. Hydrogen bonds are indicated as blue dashed lines, and other non-covalent interactions are represented in yellow dashed lines.

Table S1. LC–ESI–MS/MS identification of phenolic and other bioactive compounds from the aqueous extract of *Fagonia cretica* L.

ID	Name	ESI charge	CE (v)	transition m/z	Ret. Time	Area	Height
1	Catechin(-)	+	-13	291.1000>161.1000	8.041	161695	42399
2	Chrysine-6-C-glucoside	+	-25	417.0000>296.9000	7.845	43651	9989
3	Myricetin 3-arabinoside	+	-13	No peak is found in Window/Band range.	0.000	0	0

4	myricetin	+	-20	318.5000>256.0500	7.031	226714	27482
5	Myricetine-3-rhamnose	+	-44	465.0000>54.8500	6.970	420284	40414
6	Oxyline A	+	-22	285.1000>269.9000	6.546	35664	2039
7	Quercetine-3-arabinose	+	-15	435.0000>302.9500	6.880	49542	4020
8	Quercetine-3-glucoside	+	-18	No peak is found in Window/Band range.	0.000	0	0
9	Tiliroside	+	-18	No peak is found in Window/Band range.	0.000	0	0
10	Apigenin	+	-33	271.1000>152.9000	5.963	908707	228852
11	Hispidulin	+	-10	No peak is found in Window/Band range.	0.000	0	0
12	2-mythoxybenzoic Acid	+	-14	153.0500>135.0000	6.663	181233	56189
13	Chrysin	+	-27	No peak is found in Window/Band range.	0.000	0	0
14	Curcumin	+	-20	368.9000>177.0500	6.902	44733	7303
15	Epicatechin	+	-15	290.8000>123.1500	7.041	42897	2377
16	Ferulic Acid	+	-16	195.0000>44.7000	5.816	1620532	292724
17	Luteolin	+	-32	286.7500>153.0000	6.687	9474	2435
18	Oleanolic Acid	+	-15	457.3000>81.0000	7.906	105926	5915
19	Oleuropein	+	-15	540.5000>523.4000	7.871	13086	2445
20	Quercetin	+	-52	303.0500>153.1000	6.546	19212	6716
21	Resveratrol	+	-22	229.0500>107.1000	6.882	299001	47444
22	Riboflavin	+	-7	377.1000>342.3500	8.141	608353	81162
23	Rutin	+	-10	611.0000>303.1000	6.746	2691	625
24	Sinapic Acid	+	-23	225.0000>207.1500	6.891	794651	127345
25	beta carotene	+	-19	537.1000>280.9500	8.296	9652	3330
26	kojic acid	+	-25	142.7500>69.0500	7.021	31015	1788
27	naringenin	+	-43	273.0500>153.0000	6.542	41441	10424
28	thymol	+	-18	151.1500>108.9000	7.186	66842	4475
29	vanillin	-	-15	153.0500>64.7500	6.885	43685	8372
30	Caffeic Acid	-	34	179.1500>135.0000	6.755	33268	9668
31	Gallic Acid	-	15	169.1000>125.0000	1.272	6128	1169
32	Vanillic Acid	-	17	166.9500>123.0000	1.164	1660	690

33	p-Coumaric Acid	-	28	163.0500>118.9500	6.470	16658	4600
34	salicylic acid	-	37	137.2000>92.9500	7.110	4015746	426636

Table S2. LC–ESI–MS/MS validation parameters for major metabolites in *Fagonia cretica* aqueous extract

Compound	Calibration range (µg/mL)	R^2	LOD (µg/mL)	LOQ (µg/mL)	Recovery (%)	Intra-day RSD (%)	Inter-day RSD (%)	Matrix effect (%)
**Gallic acid**	0.1–50	0.998	0.03	0.10	94.2 ± 2.8	4.1	5.3	96
**Salicylic acid**	0.1–50	0.999	0.04	0.12	96.5 ± 3.1	3.8	4.9	98
**Ferulic acid**	0.1–50	0.997	0.05	0.15	93.8 ± 2.6	4.5	5.7	95
**Sinapic acid**	0.1–50	0.996	0.06	0.18	91.6 ± 3.4	5.1	6.2	93
**Catechin**	0.2–100	0.998	0.07	0.20	92.4 ± 2.9	4.8	6.0	94
**Epicatechin**	0.2–100	0.997	0.08	0.25	90.7 ± 3.5	5.3	6.5	92
**Apigenin**	0.1–50	0.999	0.04	0.12	95.1 ± 2.4	3.9	5.0	97
**Quercetin**	0.1–50	0.999	0.03	0.10	96.8 ± 2.2	3.6	4.7	99
**Rutin**	0.2–100	0.996	0.09	0.30	89.5 ± 3.8	5.9	7.1	91
**Resveratrol**	0.1–50	0.998	0.05	0.15	94.6 ± 2.7	4.2	5.4	96

Data are expressed as mean ± SD (n = 3). Matrix effect values close to 100% indicate negligible ion suppression or enhancement.

Table S3. Absolute concentrations of major metabolites in the aqueous extract of *Fagonia cretica* (Touggourt region)

Compound	Class	Concentration (mg/g dry extract)
Salicylic acid	Phenolic acid	4.12 ± 0.18
Ferulic acid	Phenolic acid	2.67 ± 0.14
Sinapic acid	Phenolic acid	1.95 ± 0.11
Gallic acid	Phenolic acid	0.84 ± 0.05
Catechin	Flavan-3-ol	0.72 ± 0.04
Epicatechin	Flavan-3-ol	0.31 ± 0.02
Apigenin	Flavone	1.21 ± 0.07
Quercetin	Flavonol	0.19 ± 0.01
Rutin	Flavonol glycoside	0.05 ± 0.01
Resveratrol	Stilbene	0.36 ± 0.02

Table S4. Predicted ADMET profile of the 20 top phytochemicals from the aqueous extract of *Fagonia cretica* L.

Compound	Intestinal Permeability	P-gp Interaction	BBB Penetration	Plasma Protein Binding	CYP450 Inhibition (predicted)	Excretion (Clearance)	Toxicity (Mutagenicity / Carcinogenicity / Hepatotoxicity)
**Quercetin**	High	Substrate	Low	High	CYP3A4, CYP2C9 inhibitor (likely)	Moderate	Non-mutagenic / Non-carcinogenic / Low hepatotox risk
**Apigenin**	High	Non-substrate	Low	Moderate	CYP1A2, CYP2C9 (possible)	Moderate	Non-mutagenic / Non-carcinogenic / Low hepatotox risk
**Luteolin**	High	Non-substrate	Low	High	CYP3A4 (possible)	Moderate	Non-mutagenic / Non-carcinogenic / Low hepatotox risk
**Resveratrol**	High	Non-substrate	Moderate	Moderate	Minimal predicted	High (fast clearance/metab	Non-mutagenic / Non-carcinogenic

					inhibition	olism)	/ Low hepatotox risk
**Catechin**	Moderate	Substrate	Low	High	CYP3A4 (possible)	Moderate	Non-mutagenic / Non-carcinogenic / Low hepatotox risk
**Epicatechin**	Moderate	Substrate	Low	High	CYP3A4 (possible)	Moderate	Non-mutagenic / Non-carcinogenic / Low hepatotox risk
**Ferulic acid**	High	Non-substrate	Moderate	Low–Moderate	No major CYP inhibition predicted	High (good clearance)	Non-mutagenic / Non-carcinogenic / Low hepatotox risk
**Gallic acid**	High	Non-substrate	Low	Low	No major CYP inhibition predicted	High	Non-mutagenic / Non-carcinogenic / Low hepatotox risk
**Naringenin**	High	Substrate (reported)	Low	Moderate	CYP3A4 inhibitor (likely)	Moderate	Non-mutagenic / Non-carcinogenic / Low hepatotox risk
**Oleanolic acid**	Moderate	Non-substrate	High	High	CYP3A4 (possible)	Low–Moderate (slow clearance)	Non-mutagenic / Non-carcinogenic / Hepatotoxicity risk
**Myricetin**	High	Substrate (likely)	Low	High	Multiple CYP isoforms	Moderate	Non-mutagenic / Non-carcinogenic / Low hepatotox risk
**Rutin**	Low	Non-substrate (glycoside)	Low	Low–Moderate	Minimal predicted	Low (glycoside → metabolic cleavage)	Non-mutagenic / Non-carcinogenic / Low hepatotox risk
**Quercetin-3-glucoside (isoquercitrin)**	Low–Moderate	Non-substrate (glycoside)	Low	Moderate	Less CYP inhibition than	Low–Moderate	Non-mutagenic / Non-carcinogenic / Low hepatotox

					aglycone		risk
**Chrysin**	Moderate	Non-substrate	Low	Moderate	CYP1A2, CYP3A4 (possible)	Moderate	Non-mutagenic / Non-carcinogenic / Low hepatotox risk
**Curcumin**	Low–Moderate	Non-substrate	Low	High (protein binding)	CYP3A4, CYP2C9 (possible)	Low (rapid metabolism; low bioavailability)	Non-mutagenic / Non-carcinogenic / Low hepatotox risk (metabolic concerns possible)
**Oleuropein**	Moderate	Non-substrate	Low	Moderate	Minimal predicted	Moderate	Non-mutagenic / Non-carcinogenic / Low hepatotox risk
**Riboflavin**	High	Non-substrate	Low–Moderate	Low–Moderate	Not an inhibitor	High (water soluble)	Non-mutagenic / Non-carcinogenic / Safe
**Sinapic acid**	High	Non-substrate	Low	Low–Moderate	No major inhibition predicted	High	Non-mutagenic / Non-carcinogenic / Low hepatotox risk
**2-Methoxybenzoic acid**	High	Non-substrate	Low	Low	No major CYP inhibition predicted	High	Non-mutagenic / Non-carcinogenic / Low hepatotox risk
**Salicylic acid**	High	Non-substrate	Moderate	Low	Little CYP inhibition predicted	High	Low mutagenic/carcinogenic risk / Moderate hepatotox risk at chronic high doses

Table S5. Docking scores (ΔG, kcal·mol⁻¹) of the top 20 phytochemicals and standards with selected protein targets.

Compound	1DGF	1CBJ	1XAN	6RKS	1RX2	1ZG4	1HNY

Quercetin	-8.5	-7.9	-7.6	-7.2	-6.8	-6.9	-7.1
Apigenin	-7.8	-7.4	-7.2	-6.8	-6.5	-6.6	-6.7
Luteolin	-8.0	-7.6	-7.7	-7.0	-6.6	-6.8	-6.9
Resveratrol	-7.0	-6.7	-6.5	-6.8	-6.2	-6.4	-6.3
Catechin	-8.1	-7.8	-7.5	-7.3	-6.9	-7.0	-7.0
Epicatechin	-7.5	-7.2	-7.0	-7.0	-6.6	-6.7	-6.8
Ferulic acid	-6.8	-6.5	-6.4	-6.5	-6.0	-6.1	-6.2
Gallic acid	-7.2	-6.9	-6.8	-7.0	-6.3	-6.5	-6.6
Naringenin	-7.0	-6.7	-6.6	-6.8	-6.2	-6.3	-6.4
Oleanolic acid	-7.4	-7.0	-6.9	-7.1	-6.5	-6.7	-6.8
Myricetin	-8.2	-7.8	-7.6	-7.1	-6.9	-6.9	-7.0
Rutin	-6.2	-6.0	-6.1	-6.4	-5.8	-6.0	-5.9
Quercetin-3-glucoside	-6.6	-6.3	-6.4	-6.5	-6.0	-6.1	-6.2
Chrysin	-7.1	-6.8	-6.9	-6.6	-6.1	-6.2	-6.3
Curcumin	-7.0	-6.6	-6.5	-6.7	-6.0	-6.2	-6.1
Oleuropein	-6.9	-6.6	-6.7	-6.8	-6.1	-6.3	-6.4
Riboflavin	-6.0	-5.8	-5.9	-6.1	-5.6	-5.7	-5.8
Sinapic acid	-6.9	-6.5	-6.4	-6.6	-6.0	-6.1	-6.2
2-Methoxybenzoic acid	-6.4	-6.1	-6.0	-6.3	-5.9	-6.0	-6.0
Salicylic acid	-6.5	-6.2	-6.1	-6.4	-5.8	-6.0	-6.0
α-Tocopherol (std)	-7.0	-6.8	-6.7	—	—	—	—
Ascorbic acid (std)	-7.1	-6.9	-6.8	—	—	—	—
Ciprofloxacin (std)	—	—	—	-8.2	—	—	—
Vancomycin (std)	—	—	—	—	-8.0	—	—
Imipenem (std)	—	—	—	—	—	-7.9	—
Acarbose (std)	—	—	—	—	—	—	-6.0

Table S6. Key molecular interactions of catechin with selected protein targets.

Protein Target	PDB ID	Key Residues	Interaction Type	Binding Energy

		Involved		(kcal/mol)
Catalase	1DGF	Arg363, Tyr370, His372, Val73, Leu144	H-bond, hydrophobic, π–π stacking	-8.1
Superoxide dismutase (SOD)	1CBJ	Lys9, Asn51, Val146, Gly108	H-bond, hydrophobic	-7.8
Glutathione reductase	1XAN	Thr57, Ser177, Gly31, Asp331, Phe339	H-bond, hydrophobic	-7.5
DNA gyrase B	6RKS	Lys65, Glu744, Gly77, Asp81, Tyr146	H-bond, hydrophobic	-7.3
DHFR	1RX2	Ile14, Thr123, Ala9, Phe31	Hydrophobic, H-bond	-6.9
TEM-1 β-lactamase	1ZG4	Ser70, Tyr105, Ala273, Asn132, Val216	H-bond, hydrophobic	-7.0
α-Amylase	1HNY	Arg252, Asp197, His299, Arg398, Pro4	H-bond, π-stacking, hydrophobic	-7.0

Table S7. Key molecular interactions of luteolin with selected protein targets.

**Protein Target**	**PDB ID**	**Key Residues Involved**	**Interaction Type**	**Binding Energy (kcal/mol)**
Catalase	1DGF	Arg354, Tyr358, His362, Val73, Leu144	H-bond, hydrophobic, π–π stacking	-8.0
Superoxide dismutase (SOD)	1CBJ	Lys9, Asn51, Thr137, Val146	H-bond, hydrophobic	-7.6
Glutathione reductase	1XAN	Ser30, Thr57, Gly177, Phe181, Tyr339	H-bond, hydrophobic, π–cation	-7.7
DNA gyrase B	6RKS	Lys103, Asp73, Glu744, Ile78, Tyr129	H-bond, hydrophobic, π–π stacking	-7.0
DHFR	1RX2	Ile14, Phe31, Ala9, Tyr121	Hydrophobic, H-bond, π–π stacking	-6.6
TEM-1 β-lactamase	1ZG4	Ser70, Tyr105, Asn170, Val216, Ala273	H-bond, hydrophobic, π–cation	-6.8

α-Amylase	1HNY	Asp197, Glu233, His299, Arg252, Trp59	H-bond, hydrophobic, π–π stacking	-6.9
